# Translational Genomics in Legumes Allowed Placing *In Silico* 5460 Unigenes on the Pea Functional Map and Identified Candidate Genes in *Pisum sativum* L.

**DOI:** 10.1534/g3.111.000349

**Published:** 2011-07-01

**Authors:** Amandine Bordat, Vincent Savois, Marie Nicolas, Jérome Salse, Aurélie Chauveau, Michael Bourgeois, Jean Potier, Hervé Houtin, Céline Rond, Florent Murat, Pascal Marget, Grégoire Aubert, Judith Burstin

**Affiliations:** * INRA, UMRLEG, 21065 Dijon Cedex, France; † INRA, UMR1095, 63100 Clermont-Ferrand, France

**Keywords:** *Pisum sativum*, functional consensus map, synteny, model legume species, translational genomics

## Abstract

To identify genes involved in phenotypic traits, translational genomics from highly characterized model plants to poorly characterized crop plants provides a valuable source of markers to saturate a zone of interest as well as functionally characterized candidate genes. In this paper, an integrated view of the pea genetic map was developed. A series of gene markers were mapped and their best reciprocal homologs were identified on *M. truncatula*, *L. japonicus*, soybean, and poplar pseudomolecules. Based on the syntenic relationships uncovered between pea and *M. truncatula*, 5460 pea Unigenes were tentatively placed on the consensus map. A new bioinformatics tool, http://www.thelegumeportal.net/pea_mtr_translational_toolkit, was developed that allows, for any gene sequence, to search its putative position on the pea consensus map and hence to search for candidate genes among neighboring Unigenes. As an example, a promising candidate gene for the hypernodulation mutation *nod3* in pea was proposed based on the map position of the likely homolog of *Pub1*, a *M. truncatula* gene involved in nodulation regulation. A broader view of pea genome evolution was obtained by revealing syntenic relationships between pea and sequenced genomes. Blocks of synteny were identified which gave new insights into the evolution of chromosome structure in Papillionoids and Eudicots. The power of the translational genomics approach was underlined.

Throughout history, navigators and explorers have established and progressively refined maps to discover new locations in the world. Similarly, precise genetic maps are required as a first step to locate and identify major genes as well as quantitative trait loci (QTL) involved in traits of interest. Functional genetic maps locate genes involved in physiological processes potentially underlying traits and allow identifying functional and positional candidate genes through translational genomics. Closely related species usually display syntenic regions in their genomes, in which several genes share similar map orders (Young *et al.* 2005; Zhu *et al.* 2005; Cannon *et al.* 2006; Tang *et al.* 2008). Positional candidate genes for QTL or mutations in the crop species can thus be identified in the syntenic regions of model species genomes. In legumes, excellent examples of this strategy are given by the identification of genes involved in the control of N-fixing symbiosis between pea and rhizobia through their *Medicago truncatula* or *Lotus japonicus* counterparts (Endre *et al.* 2002; Krusell *et al.* 2002; Limpens *et al.* 2003; Lévy *et al.* 2004; Stracke *et al.* 2004). In order to systematically take advantage of genomic data available in closely related species, a good knowledge of the conservation of genome organization between model and crop species should be gained.

The legume family is wide and incredibly diverse (Doyle and Luckow 2003). It includes numerous species that have been part of human diets since the dawn of agriculture and represents a valuable source of dietary proteins, as well as fibers and micronutrients (Grusak 2002; Guillon and Champ 2002; Mitchell *et al.* 2009). Pea is an important plant protein source in temperate regions of the world (10.4 M tons dry peas produced worldwide in 2009, http://faostat.fao.org/). As for other protein-rich legume crop species, rapid genetic improvement of this species is needed to meet the increasing demand for protein food sources in the world. In the Leguminosae family, total or euchromatic genome sequences are now available for three species. All three species belong to the Papilionoideae subfamily. *Medicago truncatula* is taxonomically the closest model species to cool-season legume crops such as pea, lentil, faba bean, and chickpea. They belong to the Inverted Repeat Loss Clade of the Hologalegina clade of the Papilionoidae subfamily (Doyle and Luckow 2003). The other legume model species, *Lotus japonicus*, belongs to the closely related robinioid clade of the Hologalegina clade. The third species is soybean (*Glycine max*), an economically important crop belonging to the more distant milletioid clade of the Papilionoidae subfamily. Extensive genomics resources are available for the three species and the sequencing of the euchromatic regions of the *M. truncatula* and *L. japonicus* genomes is close to completion (Cannon *et al.* 2006; Sato *et al.* 2007; Schmutz *et al.* 2010). Intra- and intergenome comparisons identified syntenic blocks between *M. truncatula* and *L. japonicus* genomes and revealed that the three legume genomes hold traces of an ancient whole genome duplication (WGD) that occurred ca. ∼59 Mya, probably after the separation of the Fabaceae from Eurosid I group including the Salicaceae (Doyle and Luckow 2003; Young *et al.* 2005; Cannon *et al.* 2006), while a more recent WGD occurred in the soybean lineage ∼13 Mya (Schmutz *et al.* 2010). Existing pea linkage maps allowed a draft evaluation of macrosynteny between pea and *M. truncatula*, *L. japonicus*, *G. max*, and other legume linkage maps (Aubert *et al.* 2006; Kaló *et al.* 2004; Choi *et al.* 2004) suggesting a significant colinearity among the genomes of these species.

In this paper, we established a new pea functional consensus map using recently developed SNP markers. This allowed us to assess in more detail the macrosynteny between pea and the three sequenced legume species by systematically searching for the best reciprocal homologs of pea Unigenes in *M. truncatula*, *L. japonicus* and *G. max* genome databases. We also used the pea genome sequence information to integrate it into previous paleo-genomics analysis in order to unravel the pea evolutionary paleo-history leading to its actual seven-chromosome structure. We used an original and robust method devoted to the identification of orthologous regions between plant genomes as well as for the detection of duplications within genomes based on integrative sequence alignment criteria combined with a statistical validation (Salse *et al.* 2009a). This method identified seven paleo-duplications in Monocots and Eudicots and allowed proposing a common ancestor with five and seven chromosomes for the Monocots and Eudicots, respectively (Salse *et al.* 2009b; Abrouk *et al.* 2010). Finally, taking advantage of the high level of synteny between pea and *M. truncatula*, we established a pea–*M. truncatula* translational toolkit allowing for any gene sequence to search its putative position on the pea consensus map, and from a position on the pea consensus map, to search for candidate genes among the neighboring placed Unigenes.

## Materials and Methods

### Development of the new pea functional consensus map

#### Plant material:

In order to increase the number of loci mapped and get more precision about their localization in our consensus map, we used six different recombinant inbred line (RIL) populations (supporting information, Table S1). We used data obtained for three RIL populations previously described: Térèse × K586 (Pop1, Laucou *et al.* 1998), Térèse × Champagne (Pop2, Loridon *et al.* 2005) and Caméor× China (Pop9, Deulvot *et al.* 2010). Furthermore, Cameor, VavD265, Ballet, three pea (*Pisum sativum* L.) lines showing variability in protein content and seed protein composition, were selected to create interconnected RIL populations by single seed descent from the crosses between Cameor × VavD265 (Pop3, 211 F6:8), Cameor × Ballet (Pop4, 207 F6:8), Ballet × VavD265 (Pop5, 211 F6:8). Leaf tissues were harvested from the F6 plant and then from a bulk of eight F7 or F8 plants for further DNA extractions. Total DNA was extracted from leaf tissues according to Dellaporta *et al.* (1983). A total of 1022 lines were used to build the consensus map.

#### Marker selection, development, and genotyping:

Microsatellite markers (Table S2) were selected based on their polymorphism and map information (Loridon *et al.* 2005) to build the framework maps of the three new populations. SSR were genotyped as described in Loridon *et al.* (2005). Moreover, 75 genes already mapped in Pop1, Pop2, Pop9 were genotyped in Pop3, Pop4, and/or Pop5 as gene-anchored bridge markers (Aubert *et al.* 2006; Deulvot *et al.* 2010, Table S2) and 34 new gene markers were developed for Pop3, Pop4 or Pop5 and presented in this paper. Additionally, 7 and 14 new gene markers scored in Pop1 (C. Rameau, personal communication) and in Pop2 (I. Lejeune-Hénaut, personal communication) were added on the consensus map. PCR amplifications of the selected genes in parents of mapping populations were carried out as described in Aubert *et al.* (2006). PCR products were directly sequenced by Millegen (Labège, France) and polymorphisms were searched. Different types of markers were then designed according to the type of polymorphism identified (INDELs, SNPs or null alleles). Genotyping conditions are summarized in Table S3. Scoring data are available at http://www.thelegumeportal.net/pea_genotype_scores.

#### Map construction:

A map was built for each RIL population as described in Aubert *et al.* (2006). Then, the consensus map was developed for the six populations (Pop1, 2, 3, 4, 5, 9). Population data were merged and the consensus map was built using CarthaGene (De Givry *et al.* 2005). The inherent difficulty of defining groups for such a large dataset was overcome by analyzing groups obtained with increasing LOD scores, from 15 to 30, using a maximum distance criterion of 30 cM. Then, markers were added to the consensus map on the basis of their relative position to common markers shared by at least two RIL populations. The map order was refined using the annealing and flips procedures of CarthaGene. The best map obtained, (*i.e.*, the map presenting the maximum log-likelihood and minimum length) was finally drawn using MapChart version 2.1 software (Voorrips 2002).

### Search for syntenic relationships between pea and *M. truncatula*, *L. japonicus*, soybean, and poplar genomes

In order to enhance the comparative mapping and assess the conservation of synteny between the *P. sativum* and *M. truncatula*, *L. japonicus*, soybean, and poplar genomes, we searched for the best reciprocal homologs of all pea gene sequences available in predicted gene sequences of the genomes of the four species.

#### Developing the pea Unigene set:

30156 pea sequences were retrieved from public databases on January 27^th^ 2010: 2227 pea CDS from public EMBLCDS database (http://www.ebi.ac.uk/embl/- ftp://ftp.ebi.ac.uk/pub/databases/embl/cds), 18552 pea EST from GenBank (dbEST), and 9377 pea EST from IPK Gatersleben (www.ipk-gatersleben.de/). Repetitive elements were masked with Repeat Masker (Smit AFA, Hubley R & Green P. /RepeatMasker Open-3.0/. 1996-2010 [http://www.repeatmasker.org]) with the parameter “-species = fabaceae” to mask both common (176) and fabaceae specific (291) repetitive elements. Low complexity regions like poly-A tails or other low quality ends were trimmed with SeqClean (Pertea *et al.* 2003). Unexpected vector sequences were removed with Seqclean using NCBI's Univec database. All the sequences under 100 bp were removed. Sequence cleaning and contig clustering were processed with EST2Uni package (Forment *et al.* 2008). Clustering has been done with the TIGR Gene Indices clustering tools (Pertea *et al.* 2003) software (TGICL) using CAP3 assembly program (Huang and Madan, 1999) with a minimum percent identity for overlaps of 95% for both programs (parameters: 'O “p95” -p 95 -n 16000'; default values: -O “p93” -p 94 -v 30 -n 1000). The annotation of the resulting Unigenes (contigs and singletons) was made based on homology to known sequences using BLASTN or tBLASTn. The e-value threshold was 1e^−15^ with a minimum identity percentage of 70%. The Unigenes were blasted against two databases in the following order: 1) the UniprotKB/Swissprot database (v57.9), 2) the predicted genes from release 3 (IMGA-gene-v3) of the Medicago Genome Sequence Consortium (MGSC) (Cannon *et al.* 2006), 3) and the *M. truncatula* Gene Index release 9 (MtGI9) from TIGR. Annotation was characterized as “very similar” if the e-value was below 1e^-20^ and “highly similar” below 1e^−50^.

#### Searching for the best reciprocal homologs of the pea sequences in *M. truncatula*, *L. japonicus*, soybean, and poplar genomes:

The search for putative orthologs of pea genes in *M. truncatula* was carried out by performing reciprocal BLASTs between the Unigenes and the *M. truncatula* sequence database containing the sequences of IMGA-gene-v3, MtGI9, and those of the BACs release 3 from MGSC. The threshold e-value was 1e^−20^ for both BLASTN. If for a given Unigene the best hit was a Medicago gene predicted sequence from IMGA-gene-v3, or an EST contig from MtGI9, a reciprocal BLASTN was performed on the pea database. If the best hit was a BAC sequence, a reciprocal BLASTN was done between a sequence containing the matching zone plus 2 kb at both 5′ and the 3′ side of the matched sequence. The best reciprocal homologs were searched using a perl script (File S1). If the best hit was the original sequence of the pea Unigene, the Medicago gene sequence was considered as the best reciprocal homolog of that pea sequence. Then, the positions of the best reciprocal homologs of pea Unigenes were searched using *M. truncatula* gene position data from the genome assembly v3 of the MGSC (http://www.medicago.org/). If the best reciprocal homolog was a BAC sequence or a predicted gene from IMGA-gene-v3, the position was given by its position on the pseudo-chromosome. If the best hit was a TC from TIGR, the position was obtained, if possible, by finding the best homolog among the predicted genes from IMGA-gene-v3 and retrieving the predicted gene position on the pseudo-chromosome. The search for putative orthologs of pea genes in *L. japonicus*, soybean (*G. max*), and poplar (*P. trichocarpa*) was achieved by reciprocal BLASTs as described for *M. truncatula*, except that only publicly available predicted gene sequences for these species were used. For *L. japonicus*, we used coding sequences from the Kazusa DNA Research Institute (http://www.kazusa.or.jp/lotus). For *G. max*, high confidence protein-coding sequences from the soybean genome were used (http://www.phytozome.net/soybean). For *P. trichocarpa*, the coding sequences were retrieved from the sequences of the *P. trichocarpa* sequencing project (http://www.phytozome.net/poplar). Finally, we compared the position of best reciprocal homologs on the pea consensus map on the one hand, and on the *M. truncatula*, *L. japonicus*, soybean and poplar pseudo-chromosomes on the other hand, using dot-plots and comparative maps. The dot-plots were built using Excel.

#### Pea genome evolution survey:

The grape, *Arabidopsis*, *Medicago*, *Lotus*, soybean, poplar, and papaya sequence databases (http://www.phytozome.org/, Abrouk *et al.* 2010) were used to perform a synteny analysis using three parameters recently defined by Salse *et al.* (2009a). These parameters increase the stringency and significance of BLAST sequence alignment by parsing BLASTX results and rebuilding HSPs (High Scoring Pairs) or pairwise sequence alignments to identify accurate paralogous and orthologous relationships. This analysis allowed searching for traces of Eudicot paleo-duplication in the pea genome.

#### The Pea_Medicago_translational_toolkit:

The “Pea_Medicago_translational_toolkit” is a cgi/perl webpage hosted on an Apache webserver (http://www.thelegumeportal.net/pea_mtr_translational_toolkit). This toolkit allows two actions: 1) searching for the putative position of a gene on the pea consensus map and 2) searching for putative candidate genes in the neighborhood of a pea genetic locus. When searching for putative gene position, the user inputs a pea sequence in the dedicated box, this sequence is BLASTed against the pea Unigene database at 1e^−20^ threshold, and the positions of the five best hits, if any, are displayed. These positions are inferred from the positions of *Medicago* best homologs of all pea Unigenes on *M. truncatula* pseudo-chromosomes relatively to *Medicago* best reciprocal homologs of the pea marker genes. When searching positional candidate genes at a locus, the user inputs either one or two pea markers, or a position in cM on the pea consensus map. The output is a list of Unigenes putatively located near the markers or the position entered by the user.

## Results

### The new *Pisum sativum L. consensus* map

**Figure 1:**
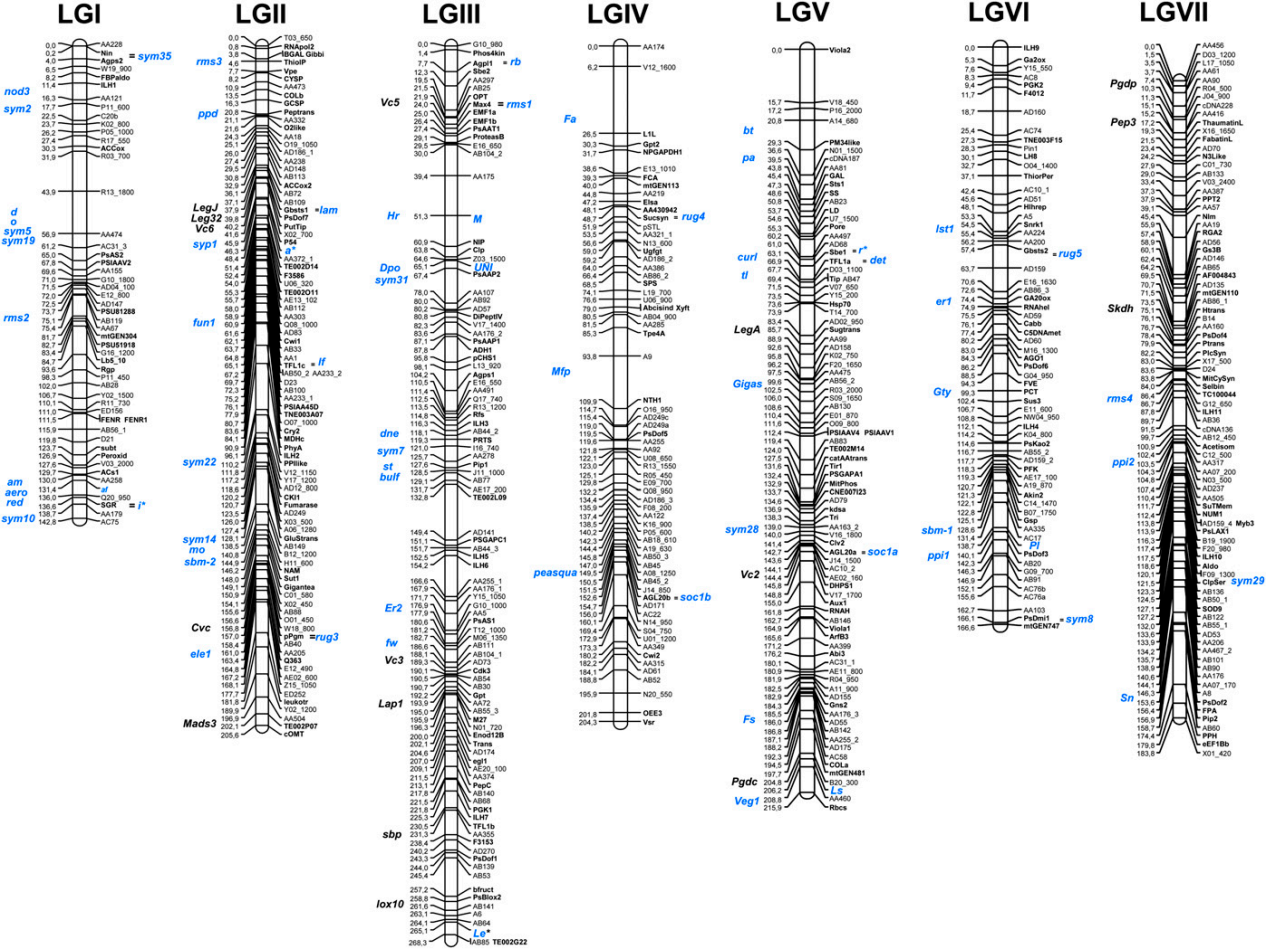
The new *Pisum sativum* L. consensus functional map. Gene markers are in bold on the right of linkage groups. Known mutations and protein or gene markers placed according to previous maps (Laucou *et al.* 1998; Hall *et al.* 1997; Weeden *et al.* 1998; Ellis and Poyser 2002; Wang *et al.* 2008; Sinjushin *et al*. 2006; Mishra *et al.* 2009; Li *et al.* 2010) are in bold on the left of linkage groups. Known mutations are in blue type. ^*^Mendel’s genes.

The map presented in Figure 1 results from a consensus mapping procedure for six different recombinant inbred line (RIL) populations (Table S1). Four new mapping populations were added to the ones used for the previous pea functional map (Aubert *et al.* 2006). Microsatellite and gene markers were mapped in populations Pop3, Pop4, and Pop5, and only gene markers were mapped in Pop9 (Deulvot *et al.* 2010). Three genomic regions displayed marker segregation distortion (Table S4): between AB33 and *Gibbi* on the top of LGII in Pop3 and Pop9, near *Le* at the bottom of LGIII in Pop5, and between AC10_1 and AD60 on LGVI in Pop3. The small distorted area found in Pop5 next to *Le* is probably due to the effect of this developmental gene driving seed number per plant (Burstin *et al.* 2007). As described in Loridon *et al.* (2005) and Aubert *et al.* (2006), marker ranks and positions were generally consistent across populations, except local rearrangements (at the top of LGI in Pop5, C20b, Agps2, AA228 and Nin are reversed; in the middle of LGII in Pop4 and of LGIII in Pop3, very close markers are reversed).

Then, the consensus map was built using bridge markers as framework markers (Table 1, Table S2). Out of the 536 markers placed on the consensus map, 56% were genotyped in at least two mapping populations, and between 16 and 154 markers were shared per pair of RIL populations (Table 1). The new consensus functional map covers 1389 cM with 97% of marker intervals below 10 cM. This map includes 3 morphological markers, 180 SSR markers, 133 RAPD, 6 RFLP, and 214 gene-based markers belonging to different functional classes (see Table S3). Fifty-one new genes were mapped in the present study and added to gene markers previously published in Aubert *et al.* (2006) and Deulvot *et al.* (2010).

**Table 1:** Number of markers shared per pair of RIL population

Population Code	Pop2	Pop3	Pop4	Pop5	Pop9
Pop1	86	71	69	71	31
Pop2		64	58	67	16
Pop3			124	154	42
Pop4				131	38
Pop5					45

This map has the advantage of incorporating markers used for different published maps (Weeden *et al.* 1998, Laucou *et al.* 1998; Choi *et al.* 2004, Loridon *et al.* 2005; Aubert *et al.* 2006; Jing *et al.* 2007; Prioul-Gervais *et al.* 2007). These links allowed us to tentatively place 48 known mutations and 15 protein or gene markers on the consensus map (Hall *et al.* 1997; Weeden *et al.* 1998; Rameau *et al.* 1998; Ellis and Poyser 2002; Hecht *et al.* 2005; Wang *et al.* 2008; Mishra *et al.* 2009; Sinjushin *et al*. 2006; Katoch *et al.* 2009; Li *et al.* 2010) (Figure 1). A total of 70 known mutations were placed on the map (Figure 1) including the 48 above-mentioned mutations and 22 mutations corresponding to mapped genes.

### Definition of the pea Unigene set

From 30,156 pea sequences retrieved from public databases, 541 have been removed and 13,381 have been trimmed after cleaning and low complexity region removal, leading to 29,615 high quality sequences. Sequences ranged from 101 to 6789 bp and had a mean length of 623 bp. The mean expressed sequence tag (EST) length was 520 bp, and the mean gene coding sequence (CDS) length was 1188 bp. The clustering produced 13,747 Unigenes: 4792 contigs and 8955 singletons. Contigs had an average length of 807 bp and an average depth of 4.3 sequences: about half of the contigs (2397) were made of two sequences, and 14% (678) were made of more than five sequences. Singletons had an average length of 525 bp. Surprisingly, 4% of singletons (346) were CDS but had no corresponding EST. A total of 10,416 (75%) of the Unigenes were annotated: 2993 with Swissprot database, 852 with Uniprot database and the remainder with the composite *M. truncatula* database. As expected, the deepest contigs, including most of ESTs, were found for the 26S ribosomal RNA, provicilin and legumin A genes.

### Identification of putative orthologs of pea Unigenes in *M. truncatula*, *L. japonicus*, soybean, and poplar sequenced genomes

**Figure 2:**
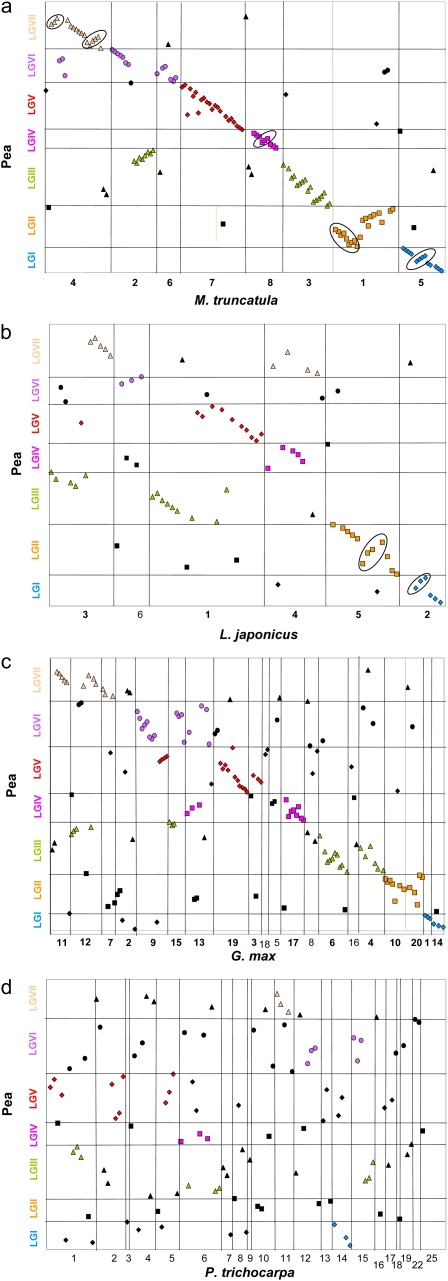
Dot-plots of syntenic relationships between the *P. sativum* linkage groups (LG) and *M. truncatula* (a), *L. japonicus* (b), *G. max* (c), and *P. trichocarpa* (d) pseudo-chromosomes. The best reciprocal homologs are placed on the dot-plot according to their position on the pea LG (Y-axis) and their position on the pseudo-chromosomes (x-axis). Synteny conservation is evidenced when homologs symbols are placed on diagonal lines. Rearrangements are circled. Syntenic blocks are highlighted according to pea LG (blue diamonds: gene mapped on PsI; yellow squares: PsII; green triangles: PsIII; pink squares: PsIV; red diamonds: PsV; purple circles: PsVI; pink triangles: LGVII); symbols are color-coded when at least three best reciprocal homologs are found between a pea LG and *M. truncatula*, *L .japonicus*, *G. max*, or *P. trichocarpa* pseudo-chromosomes.

In the search for putative *Medicago* orthologs, pea Unigenes were BLASTed on IMGA predicted genes, TIGR TC and BAC sequences (Table 2). Out of the 13,747 pea Unigene sequences, 11,166 (81%) had a unidirectional match under defined conditions and 8375 (61%) had a reciprocal best hit with the starting Unigene. Out of these 8375 best bidirectional homolog sequences, 5460 could be placed on the *M. truncatula* pseudo-chromosomes. Out of the 214 gene-based markers located on the new consensus map, 121 had a best reciprocal *M. truncatula* homolog and 19 had been defined directly from *M. truncatula* sequences. In total, 140 sequences corresponding to pea gene markers could be placed on the *M. truncatula* genome. This represents a substantial increase in the number of pea–*M. truncatula* bridges compared with Aubert *et al.* (2006) and Choi *et al.* (2004), who reported 45 and 57 links, respectively, and allowed a more precise assessment of synteny (Figure 2, Figure S1).

**Table 2:** Summary of the BLAST search for putative *Medicago* orthologs performed for the 13,747 pea Unigene sequences

	Total	IMGA Predicted Genes	TIGR TC	BAC Sequences
Unidirectional match	11,166	2631	5468	5648
% of total pea unigene	81%	19%	40%	41%
Reciprocal hits	8375	2102	4209	2064
% of total pea unigene	61%	15%	31%	15%

Shown are the total number of matched pea Unigene sequences, results of BLASTs on IMGA predicted genes, TIGR TC and BAC sequences of *Medicago* for unidirectional match under defined conditions, and reciprocal best hits with the starting gene.

A simpler method was used for searching the best reciprocal homolog in *L. japonicus*, soybean and poplar, where only predicted gene sequences were taken into account. Best reciprocal homologs were identified to pea Unigenes, and 76, 151, and 98 were placed on the *L. japonicus*, soybean, and poplar genomes, respectively (Table 3).

**Table 3:** Summary of reciprocal BLAST between pea Unigenes and *M. truncatula*, *L. japonicus*, soybean, and poplar predicted gene sequences

Species	Reciprocal Hits	Hit Rate (%)	No. of Sequences	Genome Coverage (%)	Estimated Hit Rate (%)
*M. truncatula*	5,888	43	53,425	60*^a^*	71
*L. japonicus*	5,433	40	43,051	67*^b^*	60
Soybean	6,626	48	46,430	98*^c^*	49
Poplar	2,982	22	45,778	100*^d^*	22

Shown are the number of reciprocal hits, ration of hit to total pea Unigenes, number of gene sequences available for the different species, corresponding genome sequence coverage, and estimated hit frequency if 100% of genome sequence was available.

^a^ http://www.medicago.org/genome/genome_stats.php.

^b^ Sato *et al.*, 2008.

^c^ http://www.phytozome.net/soybean.

^d^ http://www.phytozome.net/poplar.php.

### Marker synteny between the *Pisum sativum* and *M. truncatula*, *L. japonicus*, soybean and poplar genomes

**Figure 3:**
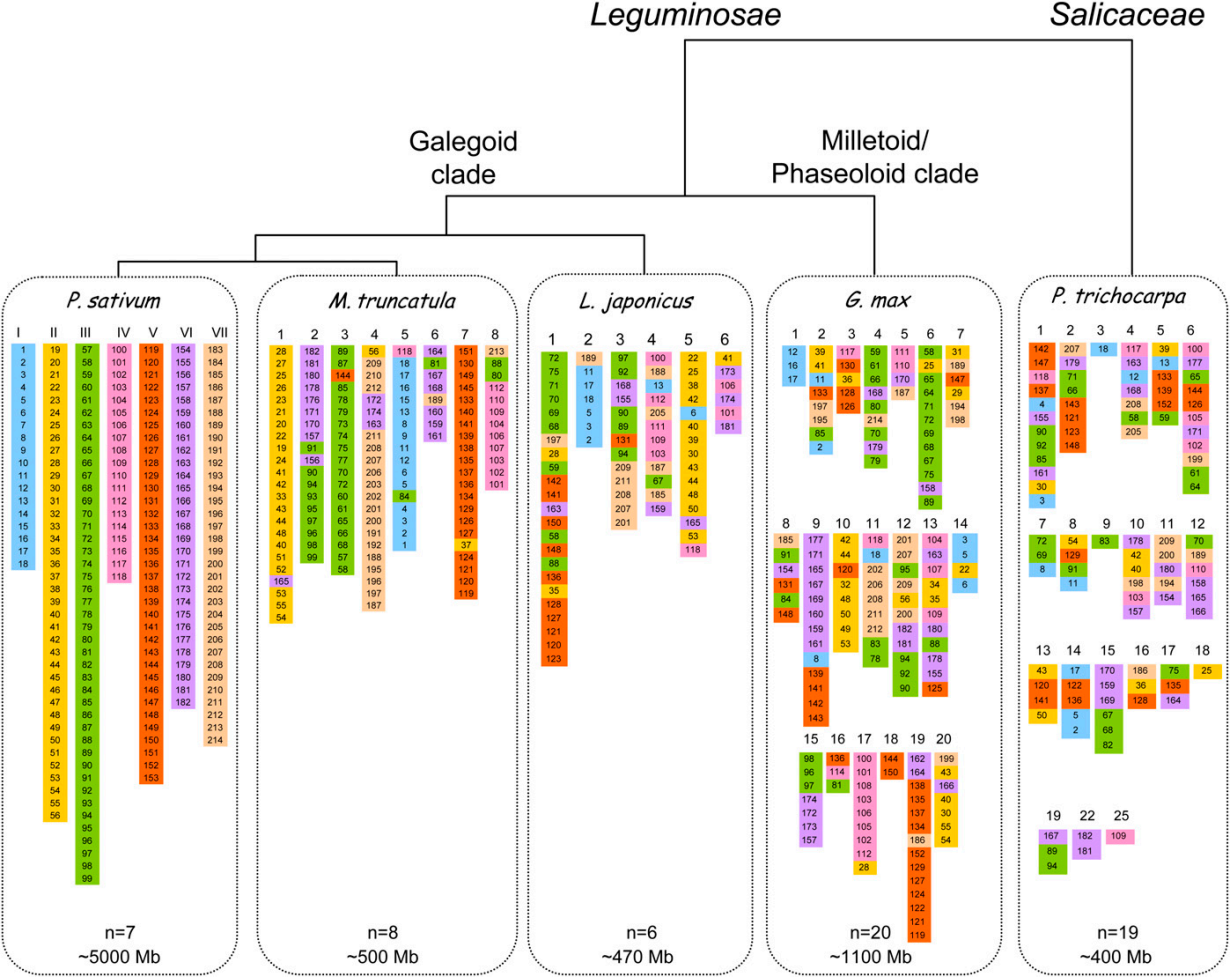
Summary view of *P. sativum*, *M. truncatula*, *L. japonicus*, soybean (G. max), and poplar (P. trichocarpa) genomes, phylogenetic relationships, and molecular characteristics. Genomes are depicted through best reciprocal homogue genes, color-coded and numbered according to the position of the *P. sativum* homolog on linkage groups of the consensus functional map.

**Figure 4:**
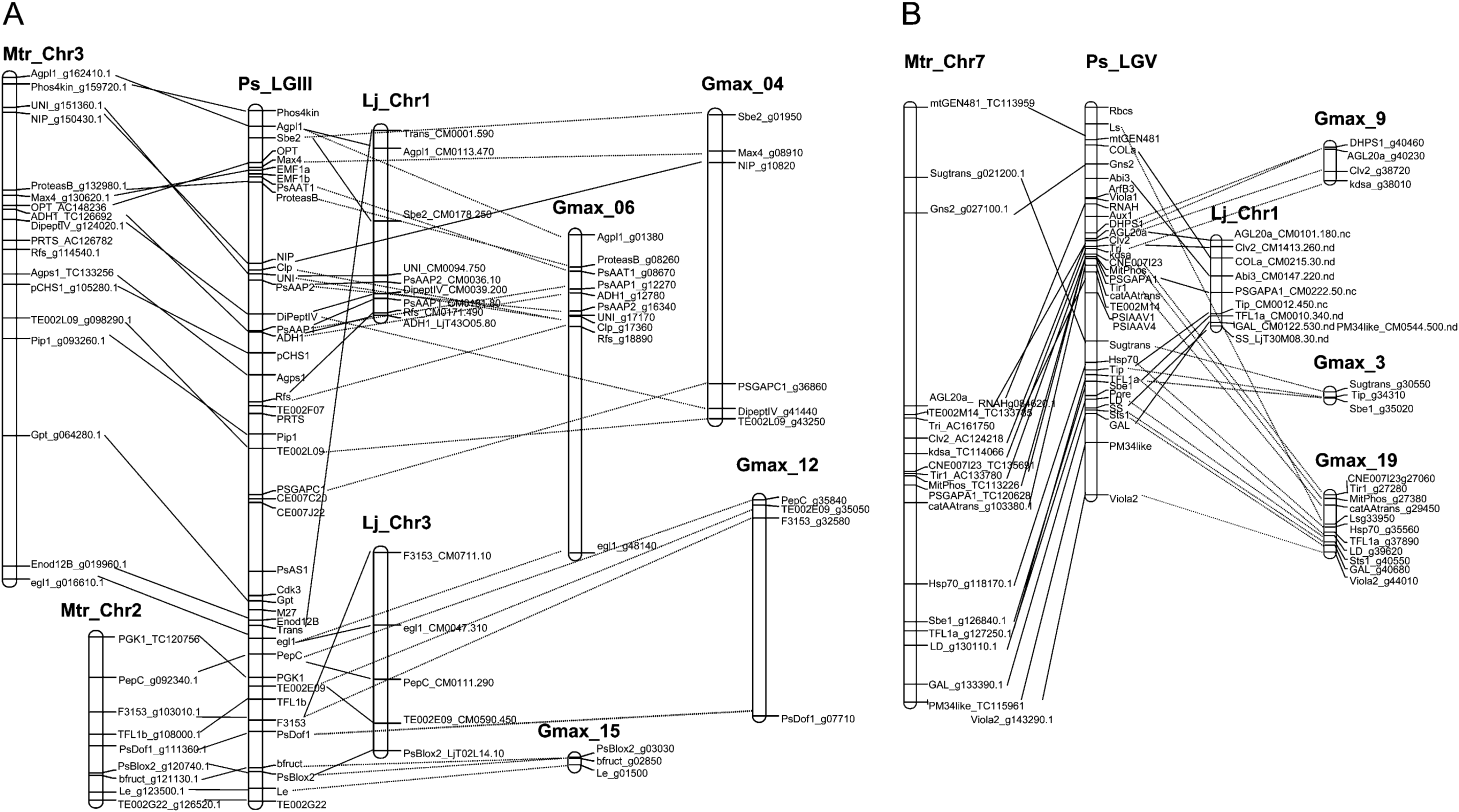
Comparative maps between *P. sativum* and *M. truncatula*, *L. japonicus*, soybean for (A) LGII and (B) LGV.

Dotplots (Figure 2) and the summary view of the chromosome relationships among the five species (Figure 3) illustrate the varying levels of conserved macrosynteny between pea and the four species analyzed. For all pea linkage groups, clear blocks of synteny linked pea and *M. truncatula* genomes with varying levels of rearrangements among the syntenic blocks. Pea linkage group I (PsI) is related to *M. truncatula* chromosome 5 (Mt5), with a small inversion of gene order in the middle, like PsIV with Mt8. PsII is related to Mt1 with an inversion at the top of PsII, and PsIII is related both to Mt2 and Mt3 with minor rearrangement along the synteny block, like PsVII with Mt4. To a lesser extent, the conservation of synteny is also striking with *L. japonicus* and soybean. For all pea linkage groups except PsVI, blocks of synteny could be identified with *L. japonicus*, with a few rearrangements (*e.g.*, the inversions in the middle of the group for PsI and PsII). As expected, synteny was more fragmented with soybean. However, clear blocks of synteny can be identified for all pea linkage groups, with from one to four syntenic blocks in soybean for each pea linkage group. Long segments of colinearity were conserved, as for example between PsV and Gm9 and 19, or PsVII with Gm11. In contrast, no clear pattern of synteny emerges from the dot-plot between pea and *P. trichocarpa* chromosomes. Comparative maps allow a closer look at the conservation of synteny blocks. Figure 4 shows syntenic regions for PsIII and PsV. While for PsV, synteny encompasses the whole linkage group, for PsIII, intra- and interchromosome rearrangements increase complexity. The pea–*M. truncatula* comparative maps (Figure S1) also inform about variations in genetic and genomic distances: for example, the longer Mt5 pseudo-molecule (42.8 Mb) corresponds to the smaller pea linkage group (PsI, 142.4 cM), suggesting a restriction of recombination for this pea chromosome.

### Pea genome evolution

**Figure 5:**
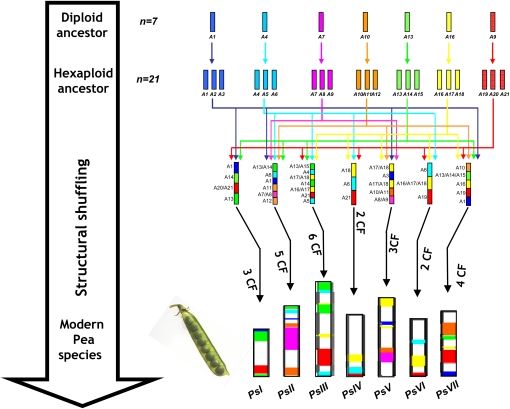
Pea genome paleo-history. The pea genome (bottom) is represented with a seven-color code to illuminate the evolution of segments from a common ancestor with seven chromosomes (A1–A7, top). Colored lines indicate the evolution of segments from a seven-chromosome common ancestor of the Eudicots to reach the modern pea genome structure. The 25 chromosomal fusions (CF) are highlighted with colored arrows. At the bottom of the figure is shown the actual pea genome structure. The ancestral shared duplications can be compared with the seven ancestral paleo-triplications reported in grape (*Vitis vinifera*, Abrouk *et al.* 2010): *V. vinifera* chromosomes are indicated by Vv, *P. sativum* linkage groups by Ps. 1Vv1-Vv14-Vv17/ PsIII-PsIV-PsV-PsVI-PsVII (yellow), Vv2-Vv12-Vv15-Vv16/PsI-PsII-PsV-PsVII (blue), Vv3-Vv4-Vv7-Vv18/PsI-PsII-PsIII-PsVII (green), Vv4-Vv9-Vv11/PsII-PsIII-PsIV-PsVI (light blue), Vv5-Vv7-Vv14/PsII-PsV (pink), Vv6-Vv8-Vv13/PsII-PsV-PsVII (brown), Vv10-Vv12-Vv19/PsI-PsIII-PsIV-PsVI-PsVII (red).

Using the alignment parameters and statistical tests described previously by Salse *et al.* (2009a,b), we analyzed the syntenic relationships between pea, grape, *Arabidopsis*, *Medicago*, *Lotus*, soybean, poplar, and papaya genomes. Using grape as the reference genome, 149 robust orthologous relationships have been identified covering 37% of the pea map. Integration of the synteny relationships established independently between pea and the seven Eudicot sequenced genomes led to the precise characterization, in pea, of the seven paleo-triplications proposed recently as the basis of the definition of seven ancestral chromosomal groups in Eudicots (Abrouk *et al.* 2010). The putative origin of shared ancestral duplications found in pea, are shown on Figure 5 using a seven color code indicating the seven common ancestor chromosomes of Eudicots. In pea, the seven ancestral shared triplications proposed recently in Eudicots (Abrouk *et al.* 2010) are thus characterized for the first time in the present paper. Based on the ancestral (γ) as well as the lineage specific (α, β) whole genome duplications reported for the Eudicots, it becomes possible to propose an evolutionary scenario that has shaped the seven pea chromosomes from the seven chromosome Eudicot ancestor and more specifically from the 21 paleo-hexaploid intermediate (Figure 5). We suggest from the 21 chromosome-intermediate ancestor at least 25 major chromosome fusions (CF) to obtain the current seven chromosome structure (Figure 5).

### A Pea-Medicago translational toolkit

Because the conservation of gene colinearity was generally good between pea and *M. truncatula*, we designed a Pea-Medicago translational toolkit based on their comparative map. If one wants to locate a gene sequence on the pea consensus map, one can enter the nucleic acid sequence of interest; this sequence will be BLASTed onto our Unigene database, andits position on the pea consensus map will be inferred from the position of this gene’s best homolog on the *M. truncatula* chromosomes. A reverse way to use this translational toolkit is to search the Unigene database for putative candidate genes in the neighborhood of a pea genetic locus of interest to the user. Of course, the validity of the predicted position will be more reliable in regions where pea–*M. truncatula* colinearity is good. It depends also on the type of gene investigated and the possibility to find the best homologs in our pea Unigene database. The translational toolkit provides a worked example. Other tools investigate synteny among legume species (Legume Information System at http://www.comparative-legumes.org/ or the *Medicago truncatula* GBrowse at http://medtr.comparative-legumes.org/gb2/gbrowse/3.5.1/). Our tool has the advantage to allow for any sequence of interest, the search of its putative position on the pea map.

## Discussion

*Pisum sativum* has been a case study for geneticists since Mendel’s pioneering studies. Since then, significant efforts have been put into the development of genetic maps for this species (Weeden *et al.* 1998; Ellis and Poyser 2002; Aubert *et al.* 2006).

### A tool for translational genomics

Many mutations have been described in pea. In 1972, Blixt (1972) published a mutation map including 169 morphological markers. Some gene markers of the present consensus map correspond to known mutations *e.g.*, the *A*, *I*, *Le* and *R* genes studied by Mendel. Other previously published mutations were placed on the functional map (Figure 1) by using bridge markers. The underlying genes responsible for many mutant phenotypes are still unknown. Our translational toolkit may help researchers 1) to find candidate genes for traits of interest, whether in pea or in a related species for which syntenic regions are identified, or 2) to define well-located gene markers in the vicinity of the gene or QTL they want to identify. For example, a root-expressed E3 ubiquitin ligase gene (PUB1) was recently shown to negatively regulate nodulation in *Medicago truncatula* (Mbengue *et al.* 2010). The translational toolkit enabled us to place *Pub1*’s closest pea homolog on the top of pea LGI, in the region of a hypernodulation mutant, *Nod3* (Gualtieri *et al.* 2002). This makes the pea ortholog of *Pub1* a very good candidate for *Nod3*.

### New features of the *P. sativum* consensus map

In this paper, we present the new consensus linkage map of *P. sativum* including 214 functional markers. Diverse functional classes are represented: development, carbohydrate metabolism, amino acid metabolism, transport, transcriptional regulation (Table S2). Because transcription factors (TF) are important key regulators of gene expression in eukaryotes and particularly seed storage protein gene expression (Meinke *et al.* 1981, Gatehouse *et al.* 1982), we mapped putative TF genes selected by homology to *M. truncatula*, *Arabidopsis thaliana*, or maize TF sequences. An OPAQUE2-LIKE gene was located on *P. sativum* LGII (PsII). In cereals, OPAQUE2 gene encodes a basic leucine zipper (bZIP) transcription factor that binds to a promoter element in the 22-kD zein genes to activate their expression (Schmidt *et al.* 1990, 1992; Ueda *et al.* 1992). Coincidentally, a legumin gene cluster was mapped in this region (Hall *et al.* 1997) as well as *Vpe*, a gene encoding for a protein involved in the maturation of storage proteins, *RNApol2* which encodes for an RNA polymerase, and *PsDof7* another transcription factor. ABSCISIC ACID-INSENSITIVE3 (ABI3) is also a seed-specific TF that has key regulatory functions during seed development, particularly in the expression of seed storage protein genes (Parcy *et al.* 1997; Kroj *et al.* 2003; Mönke *et al.* 2004) and acts in synergy with other bZIP factors (Lara *et al.* 2003). *Abi3* was mapped on LGV near four other TF genes: *ColA*, *ArfB3*, *Aux1*, and *Tir1*, and close to a previously mapped vicilin gene cluster (Hall *et al.* 1997). The genetic clustering of potentially interacting genes could favor their optimal interaction. Altogether, the functional markers located on this map might be good candidates for seed traits in pea and should be tested for their colocation with seed productivity and quality trait QTL. As in our previous work (Loridon *et al.* 2005; Aubert *et al.* 2006), we privileged easily transferable markers to allow their wide use both by academics and breeders.

### Synteny among *P. sativum*, *M. truncatula*, *L. japonicus*, and *P. trichocarpa*

In legumes, early studies reported colinearity among cool-season legume linkage maps (pea and lentil: Weeden *et al.* 1992, pea and chickpea: Simon and Muehibauer 1997). Since then, other studies have reported a high level of macrosynteny between the pea and *Medicago* genomes (Aubert *et al.* 2006; Choi *et al.* 2004; Kaló *et al.* 2004) as well as among several legume species (Choi *et al.* 2004; Zhu *et al.* 2006). With the advent of whole genome sequences, syntenic relationships can be revisited in a more detailed way in order to identify candidate genes in sequenced species for traits in crop species. In the present study, pea was put at the center of a search for syntenic relationship with four other species, from the closely related *M. truncatula* to the more distant *P. trichocarpa*. As summarized in Figure 3, the five species not only differ in their level of phylogenetic relatedness, but also in their genome size and chromosome numbers. The genome size difference is 10-fold from *L. japonicus* and *M. truncatula* small genomes (ca. 500 Mb, Sato *et al.* 2010), through soybean (ca. 1100 Mb, Schmutz *et al.* 2010), to pea (ca. 5000 Mb, Ellis and Poyser 2002). While soybean genome has undergone a recent whole genome duplication (WGD) event (ca. 13 Mya, Schmutz *et al.* 2010), the pea genome is probably the richest in retro-transposons (Ellis and Poyser 2002, Macas *et al.* 2007). Despite these marked differences, a striking conservation of gene synteny and gene order was shown in this study.

Identifying and aligning the best-in-genome homologous sequences provided a clearer picture of syntenic relationships. The highest rate of best reciprocal homolog identification for the 13,747 pea Unigenes was found for soybean (6626 putative orthologs identified, Table 3) for which close to 100% of the genome sequence is available. The second highest score was for *M. truncatula*, the closest species to pea but for which only ∼60% of the genome is covered by available sequences. The estimated number of pea Unigenes for which a putative ortholog could be expected to be found in the four other species, if 100% of the genome sequence was available (Table 3), was closely related to the phylogenetic relatedness among species, with the highest expected rate of ortholog discovery for *M. truncatula* (71%), then *L. japonicus* (60%), soybean (49%), and finally *P. trichocarpa* (22%). Similarly, decreasing levels of colinearity were correlated with higher phylogenetic distances (Figure 3). Despite the recent WGD having occurred in the soybean lineage, clear traces of synteny and coancestry were seen between the pea and soybean genomes. And although overall synteny is blurred with P. *trichocarpa*, some interesting relics merit further inspection.

A few markers broke the synteny (Figures 2 and 3). It may be that the best reciprocal homolog found in the genome of the sequenced species is not the ortholog, as the *Medicago* and *Lotus* genome sequencing are still in progress. Other possible sources of synteny breakage are gene translocation, for example following a transposition event, or ortholog gene loss making paralogs appear as best reciprocal homologs. Finally, regarding marker order rearrangements, it should be noted that the consensus map is the best statistical model obtained, but errors in marker ordering can be generated following the merge of data from different populations due to 1) translocation events as commonly reported in different pea stocks (Ellis and Poyser 2002) and 2) missing data for markers genotyped in some populations and not others, depending on their polymorphism.

Finally, this pea functional consensus map also provides a platform tool for converting markers from pea to allied species for which few functional markers are available. Through the use of *M. truncatula* as bridge species, our data relates to recent publications on lentil (Phan *et al.* 2006), white clover (George *et al.* 2008), *Phaseolus vulgaris* (McClean *et al.* 2010; Galeano *et al.* 2009; Hougaard *et al.* 2008), and chickpea (Nayak *et al.* 2010).

### Pea genome evolution

By increasing the number of mapped genes on the pea functional map, this study also significantly increased the number of bridge markers to the sequenced genomes. This allowed to put forward some hypotheses as regards to the ancient Papillionoid or Eurosid I chromosomes. The structure of the proto-papillionoid chromosome seems generally to be retained, with a few deviations. The most puzzling case involves PsIII and PsVI. When comparing pea and *M. truncatula*, it appears that PsIII is split in two in *M. truncatula*: orthologs of genes located on PsIII (Figure 3) and PsVI are found in Mt2. In *L. japonicus*, some PsIII gene orthologs are associated with PsVI gene orthologs to form part of Lj3. In *G. max*, some PsIII gene orthologs are associated with PsVI gene orthologs in Gm4, 6, 12, and 15. Finally, even in *P. trichocarpa*, some gene orthologs of PsIII and PsVI are merged for example in Pt15. Thus, it appears that PsIII and PsVI could have been part of a same chromosome before the radiation between the Leguminosae and the other Eurosid I including the Salicaceae. Two duplicated genes give another view of PsIII and PsVI evolution: while *PsAAP1* (#71 in Figure 3) and *PsAAP2* (#69) are located in neighboring positions on PsIII probably following a tandem duplication (Tegeder *et al.* 2000), the *PGK1* (#92) and *PGK2* (#182) genes (Aubert *et al.* 2006) are mapped on PsIII and PsVI.

In order to further unravel the pea genome paleo-history, we identified and characterized shared paleo-duplications based on the integration of orthologous relationships identified between the 7 pea chromosomes and the grape, *Arabidopsis*, *Medicago*, *Lotus*, soybean, poplar and papaya genomes. We proposed an evolutionary scenario that may have shaped the seven pea chromosomes from a seven chromosome paleo-hexaploid Eudicot ancestor (Abrouk *et al.* 2010). Under this hypothesis, the pea genome underwent at least one triplication event and 25 chromosome fusions (Figure 5). Interestingly, the two shorter linkage groups correspond to chromosomes having experienced few chromosomal fusion events (three for PsI and two for PsVI) during evolution, while longer linkage groups correspond to chromosomes having experienced more fusions (at least six for PsIII and four for PsV), suggesting a link between genome evolution and recombination distribution. This approach also allowed defining potential paralogous regions in the genome. For example, PsIV and PsVI are likely to share paralogous genes resulting from ancestral triplication in the eudicot ancestor. Finally, compared with the other Eudicots analyzed, pea appears to have evolved a simple genome structure, intermediate between the grape (with an ancestral-type modern genome structure) and soybean (with an intensively rearranged modern genome structure). This approach may be of general interest to identify regions potentially carrying ancient paralog genes.

In conclusion, the new consensus functional map of pea presented herein represents a significant step toward a better understanding of legume evolution of genomes and traits. Legumes are fascinating plants, establishing vital symbiosis with soil microflora, developing highly nutritive seeds, and showing an astonishing diversity of forms. Among these species, pea has a special historical interest and its genome seems to have retained a large part of the ancestral papillionoideae chromosome structure. It is thus urgent to develop more genomic resources for this species, an effort that is currently underway in different ongoing projects throughout the world.

## Supplementary Material

Supporting Information
