# Supplementary material for: Translational Genomics in Legumes Allowed Placing In Silico 5460 Unigenes on the Pea Functional Map and Identified Candidate Genes in Pisum sativum L
Source: G3 (Bethesda). 2011 Jul 1;1(2):93–103. doi: 10.1534/g3.111.000349 (PMC3276132; doi:10.1534/g3.111.000349)
Supplement: Supporting Information [file supp_1_2_93__index.html]

Supporting Information 

# Translational Genomics in Legumes Allowed Placing *In Silico* 5460 Unigenes on the Pea Functional Map and Identified Candidate Genes in *Pisum sativum* L.

## Supporting Information for Bordat*et al.*, 2011

**Files in this Data Supplement:**

- Supporting Information - Figure S1, File S1, and Tables S1-S4 (PDF, 328 KB)
- Figure S1 - Comparative maps of *P. sativum* and *M. truncatula* (PDF, 180 KB)
- File S1 - Perl script used to find best reciprocal homologues in the two output files of reciprocal BLASTs (PDF, 88 KB)
- Table S1 - Summary of recombinant inbred lines population data for the consensus mapping procedure (PDF, 40 KB)
- Table S2 - List of all markers mapped on the pea consensus functional map: type of markers, reference for genotyping conditions, and position on the pea map (Microsoft Excel, .xls, 100 KB)
- Table S3 - List of gene markers located on the consensus functional map, conditions of amplification and polymorphism screen, and functional class (Microsoft Excel, .xls, 72 KB)
- Table S4 - Segregation distortion in the mapping populations: Chi-square tests for all markers (Microsoft Excel, .xls, 96 KB)
