# Supplementary material for: Translational Genomics in Legumes Allowed Placing In Silico 5460 Unigenes on the Pea Functional Map and Identified Candidate Genes in Pisum sativum L
Source: G3 (Bethesda). 2011 Jul 1;1(2):93–103. doi: 10.1534/g3.111.000349 (PMC3276132; doi:10.1534/g3.111.000349)
Supplement: Supporting Information [file supp_1.2.93_000349SI.pdf]

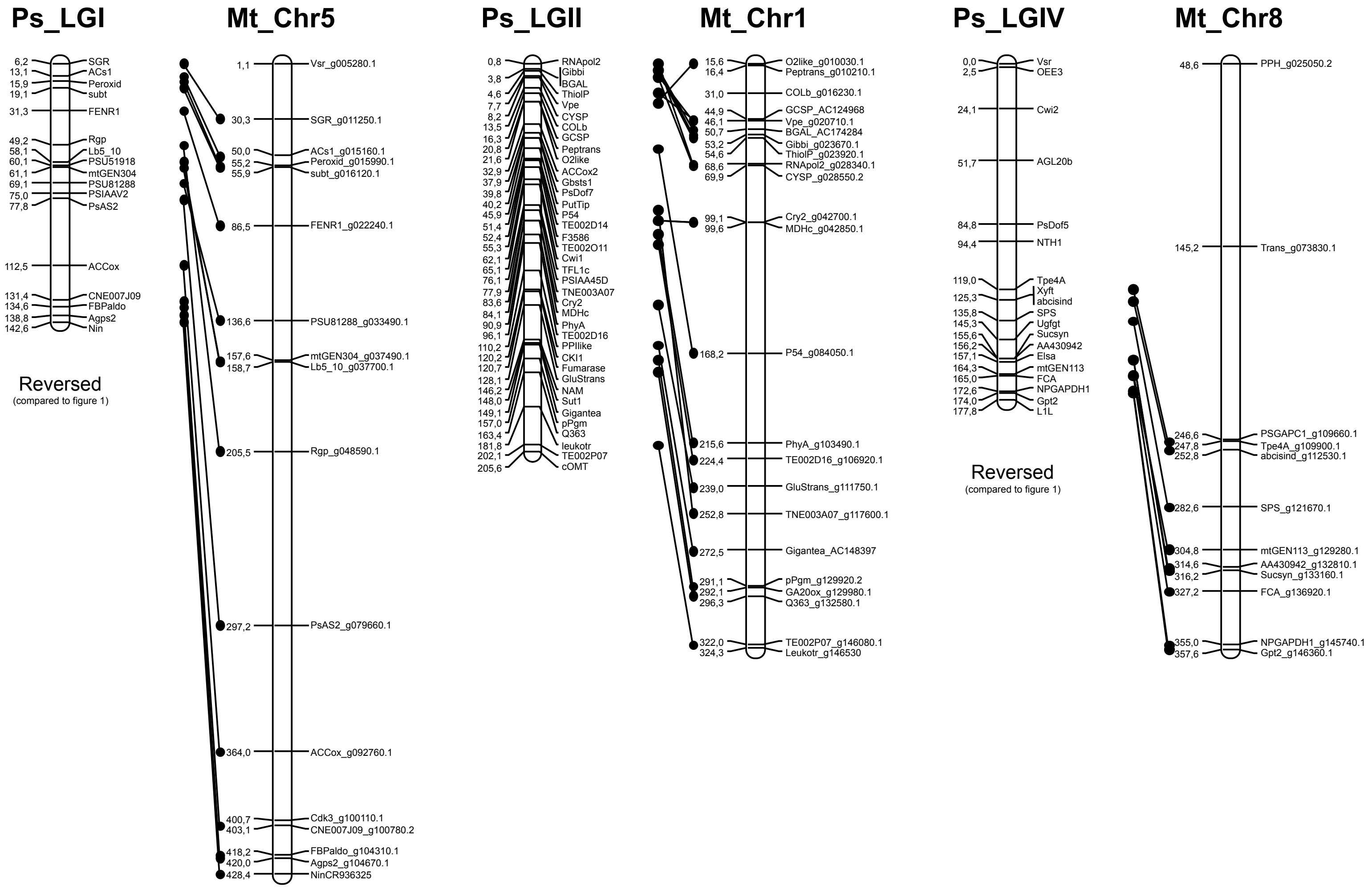
Ps_LGI
6,2 SGR
13,1 ACs1
15,9 Peroxid
19,1 subt
31,3 FENR1
49,2 Rgp
58,1 Lb5_10
60,1 PSU51918
61,1 mtGEN304
69,1 PSU81288
75,0 PSIAAV2
77,8 PsAS2
112,5 ACCox
131,4 CNE007J09
134,6 FBPaldo
138,8 Agps2
142,6 Nin
Reversed
(compared to figure 1)
Mt_Chr5
1,1 Vsr_g005280.1
30,3 SGR_g011250.1
50,0 ACs1_g015160.1
55,2 Peroxid_g015990.1
55,9 subt_g016120.1
86,5 FENR1_g022240.1
136,6 PSU81288_g033490.1
157,6 mtGEN304_g037490.1
158,7 Lb5_10_g037700.1
205,5 Rgp_g048590.1
297,2 PsAS2_g079660.1
364,0 ACCox_g092760.1
400,7 Cdk3_g100110.1
403,1 CNE007J09_g100780.2
418,2 FBPaldo_g104310.1
420,0 Agps2_g104670.1
428,4 NinCR936325
Ps_LGII
0,8 RNApol2
3,8 Gibbi
BGAL
4,6 ThiolP
7,7 Vpe
8,2 CYSP
13,5 COLb
16,3 GCSP
20,8 Peptrans
21,6 O2like
32,9 ACCox2
37,9 Gbsts1
39,8 PsDof7
40,2 PutTip
45,9 P54
51,4 TE002D14
52,4 F3586
55,3 TE002O11
62,1 Cwi1
65,1 TFL1c
76,1 PSIAA45D
77,9 TNE003A07
83,6 Cry2
84,1 MDHc
90,9 PhyA
96,1 TE002D16
110,2 PPIlike
120,2 CKI1
120,7 Fumarase
128,1 GluStrans
146,2 NAM
148,0 Sut1
149,1 Gigantea
157,0 pPgm
163,4 Q363
181,8 leukotr
202,1 TE002P07
205,6 cOMT
Mt_Chr1
15,6 O2like_g010030.1
16,4 Peptrans_g010210.1
31,0 COLb_g016230.1
44,9 GCSP_AC124968
46,1 Vpe_g020710.1
50,7 BGAL_AC174284
53,2 Gibbi_g023670.1
54,6 ThiolP_g023920.1
68,6 RNApol2_g028340.1
69,9 CYSP_g028550.2
99,1 Cry2_g042700.1
99,6 MDHc_g042850.1
168,2 P54_g084050.1
215,6 PhyA_g103490.1
224,4 TE002D16_g106920.1
239,0 GluStrans_g111750.1
252,8 TNE003A07_g117600.1
272,5 Gigantea_AC148397
291,1 pPgm_g129920.2
292,1 GA20ox_g129980.1
296,3 Q363_g132580.1
322,0 TE002P07_g146080.1
324,3 Leukotr_g146530
Ps_LGIV
0,0 Vsr
2,5 OEE3
24,1 Cwi2
51,7 AGL20b
84,8 PsDof5
94,4 NTH1
119,0 Tpe4A
Xyft
125,3 abcisind
135,8 SPS
145,3 Ugfgt
155,6 Sucsyn
156,2 AA430942
157,1 Elsa
164,3 mtGEN113
165,0 FCA
172,6 NPGAPDH1
174,0 Gpt2
177,8 L1L
Reversed
(compared to figure 1)
Mt_Chr8
48,6 PPH_g025050.2
145,2 Trans_g073830.1
246,6 PSGAPC1_g109660.1
247,8 Tpe4A_g109900.1
252,8 abcisind_g112530.1
282,6 SPS_g121670.1
304,8 mtGEN113_g129280.1
314,6 AA430942_g132810.1
316,2 Sucsyn_g133160.1
327,2 FCA_g136920.1
355,0 NPGAPDH1_g145740.1
357,6 Gpt2_g146360.1

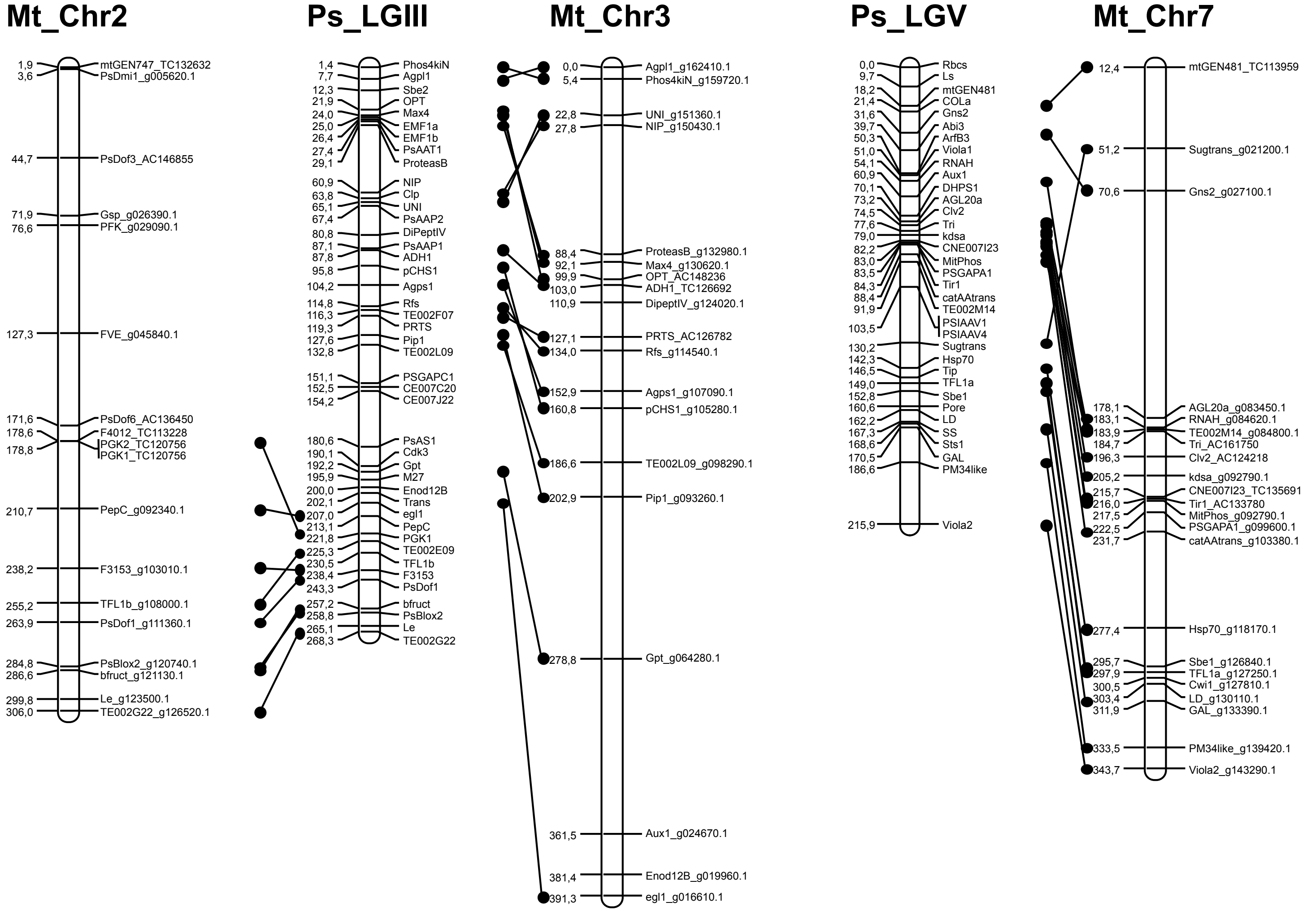
Mt_Chr2
1,9 mtGEN747_TC132632
3,6 PsDmi1_g005620.1
44,7 PsDof3_AC146855
71,9 Gsp_g026390.1
76,6 PFK_g029090.1
127,3 FVE_g045840.1
171,6 PsDof6_AC136450
178,6 F4012_TC113228
178,8 PGK2_TC120756
PGK1_TC120756
210,7 PepC_g092340.1
238,2 F3153_g103010.1
255,2 TFL1b_g108000.1
263,9 PsDof1_g111360.1
284,8 PsBlox2_g120740.1
286,6 bfruct_g121130.1
299,8 Le_g123500.1
306,0 TE002G22_g126520.1
Ps_LGIII
1,4 Phos4kiN
7,7 Agpl1
12,3 Sbe2
21,9 OPT
24,0 Max4
25,0 EMF1a
26,4 EMF1b
27,4 PsAAT1
29,1 ProteasB
60,9 NIP
63,8 Clp
65,1 UNI
67,4 PsAAP2
80,8 DiPeptIV
87,1 PsAAP1
87,8 ADH1
95,8 pCHS1
104,2 Agps1
114,8 Rfs
116,3 TE002F07
119,3 PRTS
127,6 Pip1
132,8 TE002L09
151,1 PSGAPC1
152,5 CE007C20
154,2 CE007J22
180,6 PsAS1
190,1 Cdk3
192,2 Gpt
195,9 M27
200,0 Enod12B
202,1 Trans
207,0 egl1
213,1 PepC
221,8 PGK1
225,3 TE002E09
230,5 TFL1b
238,4 F3153
243,3 PsDof1
257,2 bfruct
258,8 PsBlox2
265,1 Le
268,3 TE002G22
Mt_Chr3
0,0 Agpl1_g162410.1
5,4 Phos4kiN_g159720.1
22,8 UNI_g151360.1
27,8 NIP_g150430.1
88,4 ProteasB_g132980.1
92,1 Max4_g130620.1
99,9 OPT_AC148236
103,0 ADH1_TC126692
110,9 DipeptIV_g124020.1
127,1 PRTS_AC126782
134,0 Rfs_g114540.1
152,9 Agps1_g107090.1
160,8 pCHS1_g105280.1
186,6 TE002L09_g098290.1
202,9 Pip1_g093260.1
278,8 Gpt_g064280.1
361,5 Aux1_g024670.1
381,4 Enod12B_g019960.1
391,3 egl1_g016610.1
Ps_LGV
0,0 Rbcs
9,7 Ls
18,2 mtGEN481
21,4 COLa
31,6 Gns2
39,7 Abi3
50,3 ArfB3
51,0 Viola1
54,1 RNAH
60,9 Aux1
70,1 DHPS1
73,2 AGL20a
74,5 Clv2
77,6 Tri
79,0 kdsa
82,2 CNE007I23
83,0 MitPhos
83,5 PSGAPA1
84,3 Tir1
88,4 catAAtrans
91,9 TE002M14
103,5 PSIAAV1
PSIAAV4
130,2 Sugtrans
142,3 Hsp70
146,5 Tip
149,0 TFL1a
152,8 Sbe1
160,6 Pore
162,2 LD
167,3 SS
168,6 Sts1
170,5 GAL
186,6 PM34like
215,9 Viola2
Mt_Chr7
12,4 mtGEN481_TC113959
51,2 Sugtrans_g021200.1
70,6 Gns2_g027100.1
178,1 AGL20a_g083450.1
183,1 RNAH_g084620.1
183,9 TE002M14_g084800.1
184,7 Tri_AC161750
196,3 Clv2_AC124218
205,2 kdsa_g092790.1
215,7 CNE007I23_TC135691
216,0 Tir1_AC133780
217,5 MitPhos_g092790.1
222,5 PSGAPA1_g099600.1
231,7 catAAtrans_g103380.1
277,4 Hsp70_g118170.1
295,7 Sbe1_g126840.1
297,9 TFL1a_g127250.1
300,5 Cwi1_g127810.1
303,4 LD_g130110.1
311,9 GAL_g133390.1
333,5 PM34like_g139420.1
343,7 Viola2_g143290.1

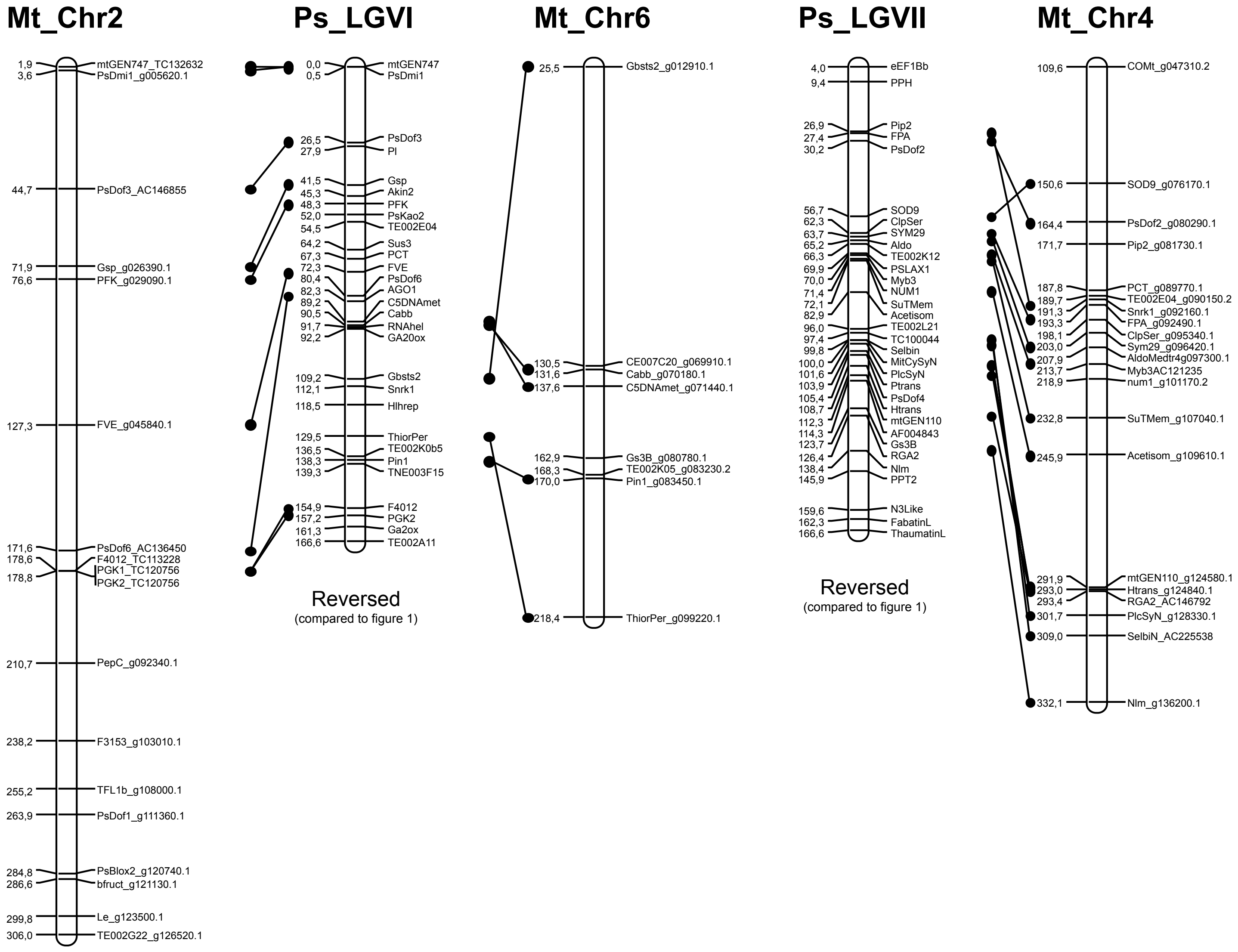

Figure S1 Comparative maps of *P. sativum* and *M. truncatula.*

File S1

Perl script used to find best reciprocal homologues in the two output files of reciprocal BLASTs.

```
#!/usr/bin/perl
#
# Copyright INRA
#
# http://www2.dijon.inra.fr/urleg/
# Vincent Savois : vincent.savois@dijon.inra.fr
#

=head1 NAME

 get_first_orthologue.pl

=head1 SYNOPSIS

 get_first_orthologue.pl blast_output_file homolog_blast_output_file > result.out

=head1  DESCRIPTION

 Search the best reciprocal homologues between pea unigenes and sequences
 from another species from two outputs of reciprocal Blasts : the database
 of the first (-d) is the input file of the other (-i) and reciprocally.

 Mandatory : the 2 file names of the reciprocal outputs.
 Standard output is the standard output (display) : please redirect to a file.
 Output file present a summary of the blast outputs : one line per reciprocal couple.

=head1 SUBROUTINES

=cut

# library
use Bio::SearchIO;


MAIN:
{
   # arguments
   my $usage          = "get_first_orthologue.pl blast_output_file homolog_blast_output_file";
   my $blastfile      = shift;
   my $homologBlastfile = shift;


   my $searchio      = new Bio::SearchIO ( -file => $blastfile,       -format => 'blast' );
   my $reci_searchio = new Bio::SearchIO ( -file => $homologBlastfile, -format => 'blast' );
   my %recis;

   while (my $reci_result = $reci_searchio->next_result)
   {
      if (my $hit = &sort_result($reci_result))
      {
         # stores only best reciprocal hits
         $recis{$reci_result->query_name}=$hit;
      }
   }

   print "requete\thit\treq reciproque\thit reciproque\tdescription\tdescription reciproque\tscore hit\tscore reciproque\te-value hit\te-value hit reciproque\n";

   #goes through the orthologues (direct sense)
   while (my $result=$searchio->next_result)
```

```
   {
      #takes only the hit we want : the best one
      if (my $hit=&sort_result($result))
      {
                  my $flag=0;
                        my $uneLigne;

         #every time, goes through all the hash of the reciprocals
                        while (my ($reci_name, $reci_hit)= each %recis)
         {
            #the comparaison is based on the names
                                    if (($hit->name eq $reci_name) && ($result->query_name eq $reci_hit->name))
            {
                                       if (my $hsp = $hit->next_hsp)
               {
                  if (my $reci_hsp = $reci_hit->next_hsp)
                  {
                           if ($reci_hsp->percent_identity > 70 && $hsp->percent_identity > 70)
                     {
                                                      $flag++;
                                                      $uneLigne = $result->query_name."\t".$hit-
>name."\t".$reci_name."\t".$reci_hit->name."\t".$hit->description."\t".$reci_hit->description."\t".$hit-
>score."\t".$reci_hit->score."\t".$hit->significance."\t".$reci_hit->significance;
                              $uneLigne = $uneLigne."\thsp\trank\t".$hsp->rank."\tident\t".$hsp-
>percent_identity."\tlength\t".$hsp->hsp_length;
                        $uneLigne = $uneLigne."\treci hsp\trank\t".$reci_hsp->rank."\tident\t".$reci_hsp-
>percent_identity."\tlength\t".$reci_hsp->hsp_length."\n";
                                                    }
                                          }
                                       }
                              }
                           }

                           if ($flag == 1)
         {
                              print $uneLigne;
                           }
                  }
   }
}

=head2

 Title        : sort_result
 Usage        : my $hit = &sort_result($result)
 Prerequisite : my $result=$searchio->next_result
 Function     : Sort hits in function of the bits value
 Returns      : the best hit
 Args         : SearchIO, next_result object

=cut

sub sort_result
{
   my $result = shift;
   my @hits;

   while (my $hit = $result->next_hit())
   {
      push @hits, $hit;
   }
   # sort by bits
   my @hits_sort = sort { $a->bits <=> $b->bits } @hits;
```

```
    $result->rewind;

    my $flag=0;
    my $temp_hit;
    while (my $hit = $result->next_hit())
    {
                if ($flag ==0)
      {
                  $temp_hit=$hit;
        $flag++;
                }
    }
    return $temp_hit;
}
```

Table S1 Summary of recombinant inbred lines population data for the consensus mapping procedure. Population code, Number of recombinant lines used in this study, number of markers genotyped, including gene markers, and SSR markers.

Pop. Code	N. Lines	N. Markers	Gene Mk	SSR Mk	Associated references*
Pop1	139	274	74	73	Laucou et al. 1998, Loridon et al. 2005, Aubert et al. 2006, Burstin et al. 2007
Pop2	164	197	59	86	Loridon et al. 2005, Aubert et al. 2007, Lejeune-Hénaut et al. 2008
Pop3	211	198	79	117	Bourgeois et al. 2011
Pop4	207	176	63	112	Bourion et al. 2010, Bourgeois et al. 2011
Pop5	211	191	77	111	Bourgeois et al. 2011
Pop9	90	92	92	0	Deulvot et al. 2010

*Associated references: Aubert et al., 2006, Theor. Appl. Genet. 112: 1024-1041; Bourgeois et al. 2011 Proteomics in press; Bourion et al. Theor. Appl. Genet. 2010, 121: 71-86; Burstin et al., 2007, Plant Physiology. 144: 768-781; Deulvot et al., 2010, BMC Genomics 11: 468; Laucou et al., 1998, Theor. Appl. Genet. 97: 905-915; Loridon et al., 2005, Theor. Appl. Genet. 111, 6: 1022-1031.

Tables S2-S4

Tables S2-S4 are available for download as Excel files at http://www.g3journal.org/lookup/suppl/doi:10.1534/g3.111.000349/-/DC1.

Table S2   List of all markers mapped on the pea consensus functional map: type of markers, reference for genotyping conditions, and position on the pea map.

Table S3: List of gene markers located on the consensus functional map, conditions of amplification and polymorphism screen, and functional class.

Table S4   Segregation distortion in the mapping populations: Chi-square tests for all markers.
