# Supplementary material for: Translational Genomics in Legumes Allowed Placing In Silico 5460 Unigenes on the Pea Functional Map and Identified Candidate Genes in Pisum sativum L
Source: G3 (Bethesda). 2011 Jul 1;1(2):93–103. doi: 10.1534/g3.111.000349 (PMC3276132; doi:10.1534/g3.111.000349)
Supplement: Supporting Information [file supp_1.2.93_FigureS1.pdf]

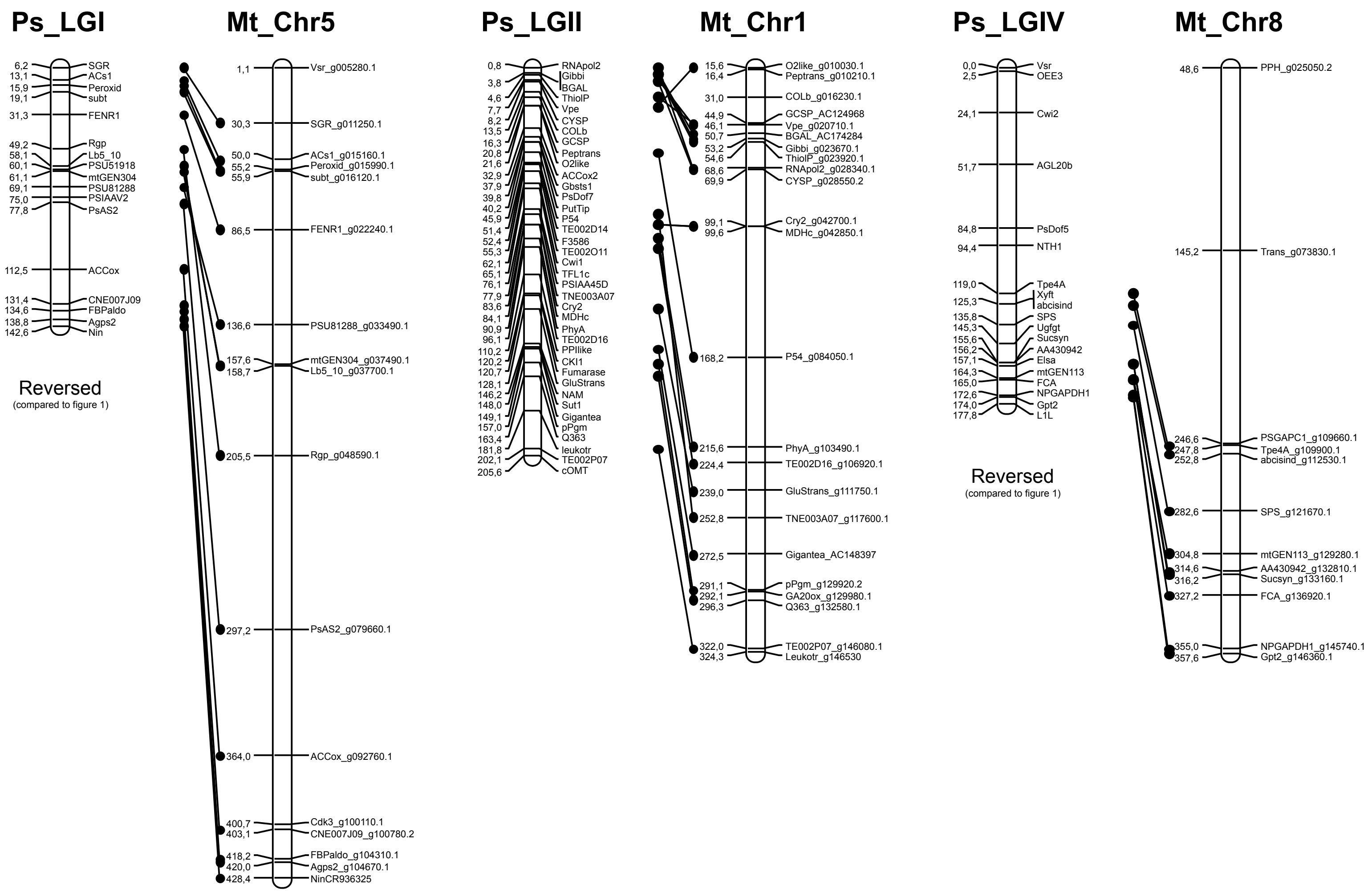
Ps_LGI
6,2 SGR
13,1 ACs1
15,9 Peroxid
19,1 subt
31,3 FENR1
49,2 Rgp
58,1 Lb5_10
60,1 PSU51918
61,1 mtGEN304
69,1 PSU81288
75,0 PSIAAV2
77,8 PsAS2
112,5 ACCox
131,4 CNE007J09
134,6 FBPaldo
138,8 Agps2
142,6 Nin
Reversed
(compared to figure 1)
Mt_Chr5
1,1 Vsr_g005280.1
30,3 SGR_g011250.1
50,0 ACs1_g015160.1
55,2 Peroxid_g015990.1
55,9 subt_g016120.1
86,5 FENR1_g022240.1
136,6 PSU81288_g033490.1
157,6 mtGEN304_g037490.1
158,7 Lb5_10_g037700.1
205,5 Rgp_g048590.1
297,2 PsAS2_g079660.1
364,0 ACCox_g092760.1
400,7 Cdk3_g100110.1
403,1 CNE007J09_g100780.2
418,2 FBPaldo_g104310.1
420,0 Agps2_g104670.1
428,4 NinCR936325
Ps_LGII
0,8 RNApol2
3,8 Gibbi
4,6 BGAL
7,7 ThiolP
8,2 Vpe
13,5 CYSP
16,3 COLb
20,8 GCSP
21,6 Peptrans
32,9 O2like
37,9 ACCox2
39,8 Gbsts1
40,2 PsDof7
45,9 PutTip
51,4 P54
52,4 TE002D14
55,3 F3586
62,1 TE002O11
65,1 Cwi1
76,1 TFL1c
77,9 PSIAA45D
83,6 TNE003A07
84,1 Cry2
90,9 MDHc
96,1 PhyA
110,2 TE002D16
120,2 PPIlike
120,7 CKI1
128,1 Fumarase
146,2 GluStrans
148,0 NAM
149,1 Sut1
157,0 Gigantea
163,4 pPgm
181,8 Q363
202,1 leukotr
205,6 TE002P07
cOMT
Mt_Chr1
15,6 O2like_g010030.1
16,4 Peptrans_g010210.1
31,0 COLb_g016230.1
44,9 GCSP_AC124968
46,1 Vpe_g020710.1
50,7 BGAL_AC174284
53,2 Gibbi_g023670.1
54,6 ThiolP_g023920.1
68,6 RNApol2_g028340.1
69,9 CYSP_g028550.2
99,1 Cry2_g042700.1
99,6 MDHc_g042850.1
168,2 P54_g084050.1
215,6 PhyA_g103490.1
224,4 TE002D16_g106920.1
239,0 GluStrans_g111750.1
252,8 TNE003A07_g117600.1
272,5 Gigantea_AC148397
291,1 pPgm_g129920.2
292,1 GA20ox_g129980.1
296,3 Q363_g132580.1
322,0 TE002P07_g146080.1
324,3 Leukotr_g146530
Ps_LGIV
0,0 Vsr
2,5 OEE3
24,1 Cwi2
51,7 AGL20b
84,8 PsDof5
94,4 NTH1
119,0 Tpe4A
125,3 Xyft
135,8 abcisind
145,3 SPS
155,6 Ugfgt
156,2 Sucsyn
157,1 AA430942
164,3 Elsa
165,0 mtGEN113
172,6 FCA
174,0 NPGAPDH1
177,8 Gpt2
L1L
Reversed
(compared to figure 1)
Mt_Chr8
48,6 PPH_g025050.2
145,2 Trans_g073830.1
246,6 PSGAPC1_g109660.1
247,8 Tpe4A_g109900.1
252,8 abcisind_g112530.1
282,6 SPS_g121670.1
304,8 mtGEN113_g129280.1
314,6 AA430942_g132810.1
316,2 Sucsyn_g133160.1
327,2 FCA_g136920.1
355,0 NPGAPDH1_g145740.1
357,6 Gpt2_g146360.1

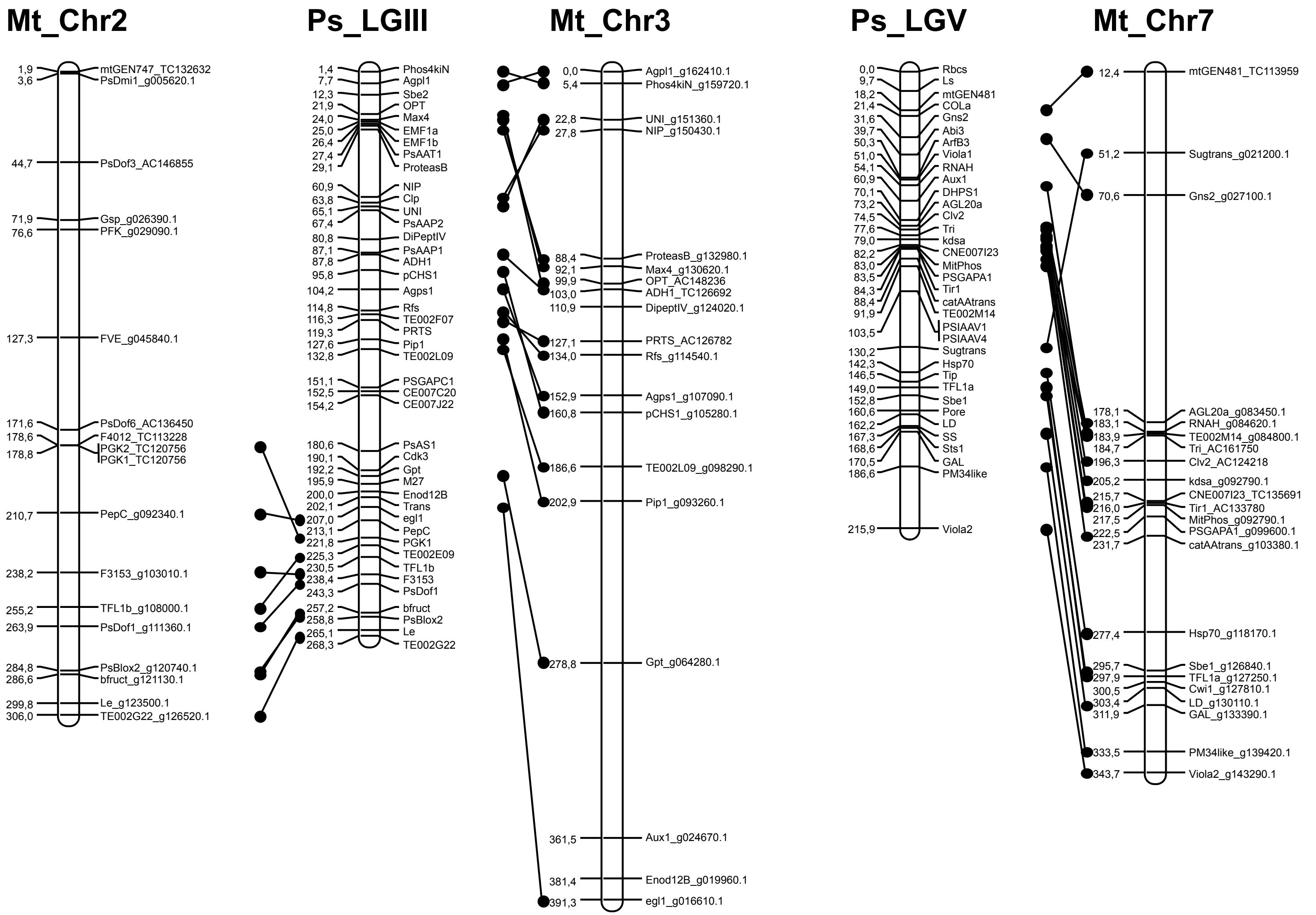

Mt_Chr2
1,9 mtGEN747_TC132632
3,6 PsDmi1_g005620.1
44,7 PsDof3_AC146855
71,9 Gsp_g026390.1
76,6 PFK_g029090.1
127,3 FVE_g045840.1
171,6 PsDof6_AC136450
178,6 F4012_TC113228
178,8 PGK2_TC120756
PGK1_TC120756
210,7 PepC_g092340.1
238,2 F3153_g103010.1
255,2 TFL1b_g108000.1
263,9 PsDof1_g111360.1
284,8 PsBlox2_g120740.1
286,6 bfruct_g121130.1
299,8 Le_g123500.1
306,0 TE002G22_g126520.1
Ps_LGIII
1,4 Phos4kiN
7,7 Agpl1
12,3 Sbe2
21,9 OPT
24,0 Max4
25,0 EMF1a
26,4 EMF1b
27,4 PsAAT1
29,1 ProteasB
60,9 NIP
63,8 Clp
65,1 UNI
67,4 PsAAP2
80,8 DiPeptIV
87,1 PsAAP1
87,8 ADH1
95,8 pCHS1
104,2 Agps1
114,8 Rfs
116,3 TE002F07
119,3 PRTS
127,6 Pip1
132,8 TE002L09
151,1 PSGAPC1
152,5 CE007C20
154,2 CE007J22
180,6 PsAS1
190,1 Cdk3
192,2 Gpt
195,9 M27
200,0 Enod12B
202,1 Trans
207,0 egl1
213,1 PepC
221,8 PGK1
225,3 TE002E09
230,5 TFL1b
238,4 F3153
243,3 PsDof1
257,2 bfruct
258,8 PsBlox2
265,1 Le
268,3 TE002G22
Mt_Chr3
0,0 Agpl1_g162410.1
5,4 Phos4kiN_g159720.1
22,8 UNI_g151360.1
27,8 NIP_g150430.1
88,4 ProteasB_g132980.1
92,1 Max4_g130620.1
99,9 OPT_AC148236
103,0 ADH1_TC126692
110,9 DipeptIV_g124020.1
127,1 PRTS_AC126782
134,0 Rfs_g114540.1
152,9 Agps1_g107090.1
160,8 pCHS1_g105280.1
186,6 TE002L09_g098290.1
202,9 Pip1_g093260.1
278,8 Gpt_g064280.1
361,5 Aux1_g024670.1
381,4 Enod12B_g019960.1
391,3 egl1_g016610.1
Ps_LGV
0,0 Rbcs
9,7 Ls
18,2 mtGEN481
21,4 COLa
31,6 Gns2
39,7 Abi3
50,3 ArfB3
51,0 Viola1
54,1 RNAH
60,9 Aux1
70,1 DHPS1
73,2 AGL20a
74,5 Clv2
77,6 Tri
79,0 kdsa
82,2 CNE007I23
83,0 MitPhos
83,5 PSGAPA1
84,3 Tir1
88,4 catAAtrans
91,9 TE002M14
103,5 PSIAAV1
PSIAAV4
130,2 Sugtrans
142,3 Hsp70
146,5 Tip
149,0 TFL1a
152,8 Sbe1
160,6 Pore
162,2 LD
167,3 SS
168,6 Sts1
170,5 GAL
186,6 PM34like
215,9 Viola2
Mt_Chr7
12,4 mtGEN481_TC113959
51,2 Sugtrans_g021200.1
70,6 Gns2_g027100.1
178,1 AGL20a_g083450.1
183,1 RNAH_g084620.1
183,9 TE002M14_g084800.1
184,7 Tri_AC161750
196,3 Clv2_AC124218
205,2 kdsa_g092790.1
215,7 CNE007I23_TC135691
216,0 Tir1_AC133780
217,5 MitPhos_g092790.1
222,5 PSGAPA1_g099600.1
231,7 catAAtrans_g103380.1
277,4 Hsp70_g118170.1
295,7 Sbe1_g126840.1
297,9 TFL1a_g127250.1
300,5 Cwi1_g127810.1
303,4 LD_g130110.1
311,9 GAL_g133390.1
333,5 PM34like_g139420.1
343,7 Viola2_g143290.1

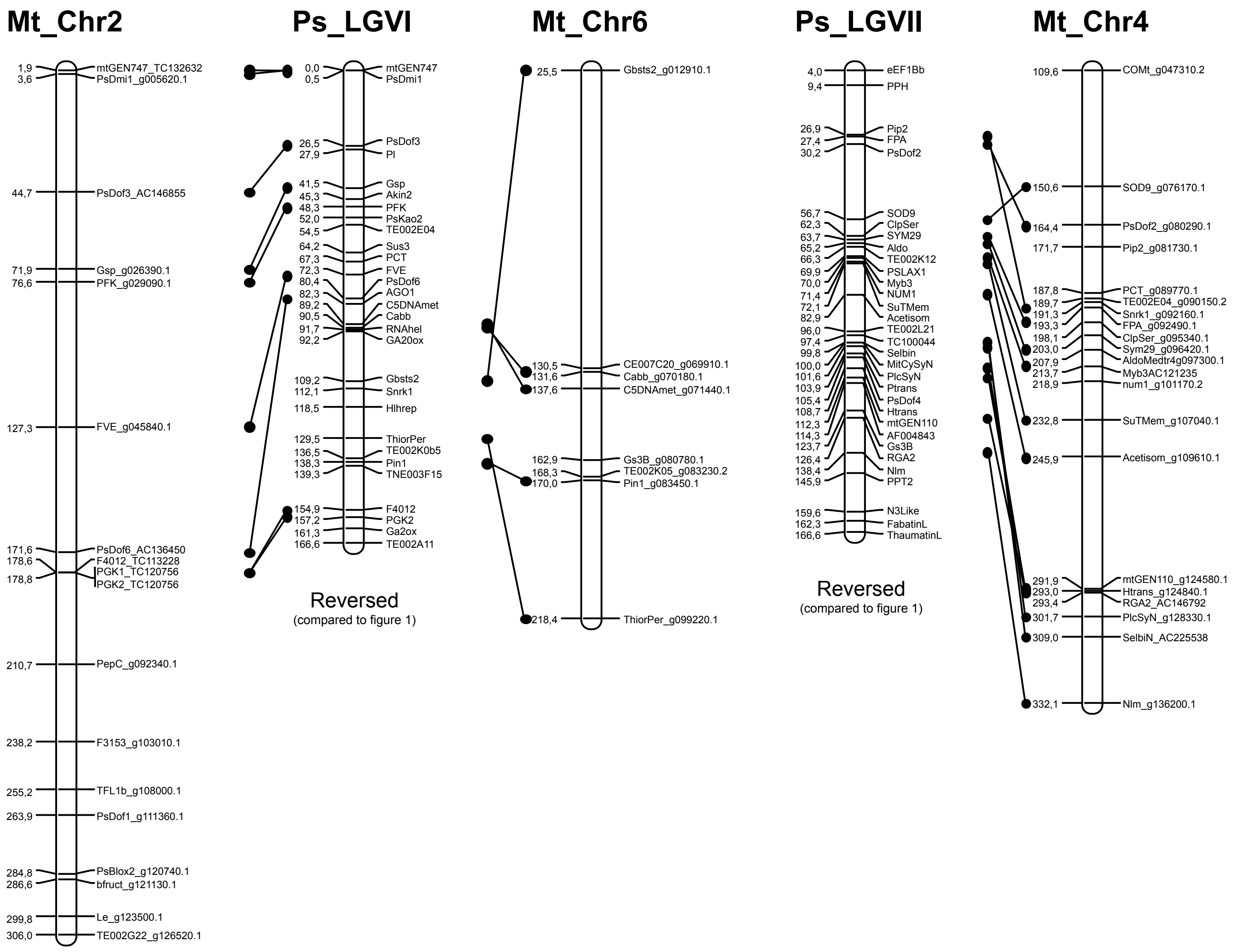

Figure S1 Comparative maps of *P. sativum* and *M. truncatula.*
