# Supplementary material for: Translational Genomics in Legumes Allowed Placing In Silico 5460 Unigenes on the Pea Functional Map and Identified Candidate Genes in Pisum sativum L
Source: G3 (Bethesda). 2011 Jul 1;1(2):93–103. doi: 10.1534/g3.111.000349 (PMC3276132; doi:10.1534/g3.111.000349)
Supplement: Supporting Information [file supp_1.2.93_FileS1.pdf]

**File S1**

**Perl script used to find best reciprocal homologues in the two output files of reciprocal BLASTs.**

```
#!/usr/bin/perl
#
# Copyright INRA
#
# http://www2.dijon.inra.fr/urleg/
# Vincent Savois : vincent.savois@dijon.inra.fr
#

=head1 NAME

 get_first_orthologue.pl

=head1 SYNOPSIS

 get_first_orthologue.pl blast_output_file homolog_blast_output_file > result.out

=head1  DESCRIPTION

 Search the best reciprocal homologues between pea unigenes and sequences
 from another species from two outputs of reciprocal Blasts : the database
 of the first (-d) is the input file of the other (-i) and reciprocally.

 Mandatory : the 2 file names of the reciprocal outputs.
 Standard output is the standard output (display) : please redirect to a file.
 Output file present a summary of the blast outputs : one line per reciprocal couple.

=head1 SUBROUTINES

=cut

# library
use Bio::SearchIO;


MAIN:
{
   # arguments
   my $usage          = "get_first_orthologue.pl blast_output_file homolog_blast_output_file";
   my $blastfile       = shift;
   my $homologBlastfile = shift;


   my $searchio      = new Bio::SearchIO ( -file => $blastfile,       -format => 'blast' );
   my $reci_searchio = new Bio::SearchIO ( -file => $homologBlastfile, -format => 'blast' );
   my %recis;

   while (my $reci_result = $reci_searchio->next_result)
   {
      if (my $hit = &sort_result($reci_result))
      {
         # stores only best reciprocal hits
         $recis{$reci_result->query_name}=$hit;
      }
   }

   print "requete\thit\treq reciproque\thit reciproque\tdescription\tdescription reciproque\tscore hit\tscore reciproque\te-value hit\te-value hit reciproque\n";

   #goes through the orthologues (direct sense)
   while (my $result=$searchio->next_result)
```

```
   {
      #takes only the hit we want : the best one
      if (my $hit=&sort_result($result))
      {
                  my $flag=0;
                           my $uneLigne;

         #every time, goes through all the hash of the reciprocals
                           while (my ($reci_name, $reci_hit)= each %recis)
         {
            #the comparaison is based on the names
                                       if (($hit->name eq $reci_name) && ($result->query_name eq $reci_hit->name))
            {
                                          if (my $hsp = $hit->next_hsp)
               {
                  if (my $reci_hsp = $reci_hit->next_hsp)
                  {
                              if ($reci_hsp->percent_identity > 70 && $hsp->percent_identity > 70)
                     {
                                                            $flag++;
                                                            $uneLigne = $result->query_name."\t".$hit-
>name."\t".$reci_name."\t".$reci_hit->name."\t".$hit->description."\t".$reci_hit->description."\t".$hit-
>score."\t".$reci_hit->score."\t".$hit->significance."\t".$reci_hit->significance;
                              $uneLigne = $uneLigne."\thsp\trank\t".$hsp->rank."\tident\t".$hsp-
>percent_identity."\tlength\t".$hsp->hsp_length;
                        $uneLigne = $uneLigne."\treci hsp\trank\t".$reci_hsp->rank."\tident\t".$reci_hsp-
>percent_identity."\tlength\t".$reci_hsp->hsp_length."\n";
                                                          }
                                                }
                                             }
                                    }
                                 }

                                 if ($flag == 1)
         {
                                    print $uneLigne;
                                 }
               }
   }
}

=head2

 Title        : sort_result
 Usage        : my $hit = &sort_result($result)
 Prerequisite : my $result=$searchio->next_result
 Function     : Sort hits in function of the bits value
 Returns      : the best hit
 Args         : SearchIO, next_result object

=cut

sub sort_result
{
   my $result = shift;
   my @hits;

   while (my $hit = $result->next_hit())
   {
      push @hits, $hit;
   }
   # sort by bits
   my @hits_sort = sort { $a->bits <=> $b->bits } @hits;
```

```
    $result->rewind;

    my $flag=0;
    my $temp_hit;
    while (my $hit = $result->next_hit())
    {
                if ($flag ==0)
       {
                   $temp_hit=$hit;
          $flag++;
                }
    }
    return $temp_hit;
}
```
