# Supplementary material for: Translational Genomics in Legumes Allowed Placing In Silico 5460 Unigenes on the Pea Functional Map and Identified Candidate Genes in Pisum sativum L
Source: G3 (Bethesda). 2011 Jul 1;1(2):93–103. doi: 10.1534/g3.111.000349 (PMC3276132; doi:10.1534/g3.111.000349)
Supplement: Supporting Information [file supp_1.2.93_TableS1.pdf]

Table S1 Summary of recombinant inbred lines population data for the consensus mapping procedure. Population code, Number of recombinant lines used in this study, number of markers genotyped, including gene markers, and SSR markers.

Pop. Code	N. Lines	N. Markers	Gene Mk	SSR Mk	Associated references*
Pop1	139	274	74	73	Laucou et al. 1998, Loridon et al. 2005, Aubert et al. 2006, Burstin et al. 2007
Pop2	164	197	59	86	Loridon et al. 2005, Aubert et al. 2007, Lejeune-Hénaut et al. 2008
Pop3	211	198	79	117	Bourgeois et al. 2011
Pop4	207	176	63	112	Bourion et al. 2010, Bourgeois et al. 2011
Pop5	211	191	77	111	Bourgeois et al. 2011
Pop9	90	92	92	0	Deulvot et al. 2010

*Associated references: Aubert et al., 2006, Theor. Appl. Genet. 112: 1024-1041; Bourgeois et al. 2011 Proteomics in press; Bourion et al. Theor. Appl. Genet. 2010, 121: 71-86; Burstin et al., 2007, Plant Physiology. 144: 768-781; Deulvot et al., 2010, BMC Genomics 11: 468; Laucou et al., 1998, Theor. Appl. Genet. 97: 905-915; Loridon et al., 2005, Theor. Appl. Genet. 111, 6: 1022-1031.
